# Potential impact of celiac disease genetic risk factors on T cell receptor signaling in gluten-specific CD4+ T cells

**DOI:** 10.1038/s41598-021-86612-5

**Published:** 2021-04-29

**Authors:** Olivier B. Bakker, Aarón D. Ramírez-Sánchez, Zuzanna A. Borek, Niek de Klein, Yang Li, Rutger Modderman, Yvonne Kooy-Winkelaar, Marie K. Johannesen, Filomena Matarese, Joost H. A. Martens, Vinod Kumar, Jeroen van Bergen, Shuo-Wang Qiao, Knut E. A. Lundin, Ludvig M. Sollid, Frits Koning, Cisca Wijmenga, Sebo Withoff, Iris H. Jonkers

**Affiliations:** 1 Department of Genetics, University Medical Center Groningen, University of Groningen, Groningen, The Netherlands; 2 Department of Immunohematology and Blood Transfusion, Leiden University Medical Center, Leiden, The Netherlands; 3 K.G. Jebsen Coeliac Disease Research Centre, Institute of Clinical Medicine, University of Oslo, Oslo, Norway; 4 Department of Immunology, Oslo University Hospital, Rikshospitalet, Oslo, Norway; 5 Department of Molecular Biology, Nijmegen Centre for Molecular Life Sciences, Radboud University, Nijmegen, The Netherlands; 6 Department of Internal Medicine and Radboud Center for Infectious Diseases, Radboud University Medical Center, Nijmegen, The Netherlands; 7 Nitte (Deemed to be University), Division of Infectious Diseases, Nitte University Centre for Science Education and Research (NUCSER), Paneer Campus, Deralakatte, Mangaluru, 575018 India; 8 Department of Gastroenterology, Oslo University Hospital, Rikshospitalet, Oslo, Norway

**Keywords:** Coeliac disease, Epigenetics in immune cells, CD4-positive T cells, Coeliac disease, Gene expression profiling, Proteomic analysis

## Abstract

Celiac disease is an auto-immune disease in which an immune response to dietary gluten leads to inflammation and subsequent atrophy of small intestinal villi, causing severe bowel discomfort and malabsorption of nutrients. The major instigating factor for the immune response in celiac disease is the activation of gluten-specific CD4+ T cells expressing T cell receptors that recognize gluten peptides presented in the context of HLA-DQ2 and DQ8. Here we provide an in-depth characterization of 28 gluten-specific T cell clones. We assess their transcriptional and epigenetic response to T cell receptor stimulation and link this to genetic factors associated with celiac disease. Gluten-specific T cells have a distinct transcriptional profile that mostly resembles that of Th1 cells but also express cytokines characteristic of other types of T-helper cells. This transcriptional response appears not to be regulated by changes in chromatin state, but rather by early upregulation of transcription factors and non-coding RNAs that likely orchestrate the subsequent activation of genes that play a role in immune pathways. Finally, integration of chromatin and transcription factor binding profiles suggest that genes activated by T cell receptor stimulation of gluten‑specific T cells may be impacted by genetic variation at several genetic loci associated with celiac disease.

## Introduction

In celiac disease (CeD), cereal-derived gluten peptides penetrate the small intestinal barrier, are subsequently modified by tissue trans-glutaminase 2 (TG2), then presented by HLA-DQ2- or HLA-DQ8-positive antigen-presenting cells (APCs) to gluten-specific CD4+ T-helper cells (gsTcells)^1^. This leads to robust activation of gsTcells that subsequently stimulate B cells to start producing auto antibodies to TG2 and deamidated gluten peptides^1,2^ and activate CD8+ intraepithelial lymphocytes (IELs) to attack intestinal epithelial cells, leading to the villous atrophy that is characteristic of CeD. GsTcells are only found persistently in CeD patients^3,4^ and can induce villous atrophy in patients upon gluten ingestion even after these individuals have been on a gluten-free diet for years^5^. Activation of gsTcells is thus central to CeD onset and pathology.

GsTcells have been shown to secrete many signaling molecules upon stimulation, including interleukin (IL)-2, IL-4, IL-6, IL-8, IL-10, IL-21, CD40LG, IFNγ and TNF, and are often classified to be of type 1 helper class^6–12^. GsTcells uniquely express IL-21 and CXCL13, as well as several other markers characteristic of follicular and regulatory T cells^13^. IL-21 and CXCL13, together with CD40LG and IL-4, play an important role in the interaction, differentiation and activation of T cells and plasma B cells^13–16^. Cytokines secreted by gsTcells are also important for activation and proliferation of IELs, in combination with IL-15, a cytokine important in CeD etiology that is produced by IELs^17–20^. GsTcells are thus central in the response to gluten peptides that leads to inflammation, anti-TG2 antibody production and villous atrophy in CeD.

To date, 43 genetic risk factors have been associated with CeD^21–23^, the most important being the HLA haplotypes HLA-DQ2 and -DQ8. While the role of HLA-DQ2 and -DQ8 in CeD is well defined^24–26^, the contribution of the non-HLA CeD risk-loci is mostly unclear. More than 95% of the single nucleotide polymorphisms (SNPs) associated with CeD are located in the non-coding genome and presumably deregulate genes important for CeD etiology^27^. Enrichment analysis of the CeD SNPs in regulatory regions suggests that CD4+ T cells are the major cell type affected by genetic risk factors^28–30^. Moreover, pathway and *cis*-eQTL analyses of genes in CeD loci suggest that they affect T cell receptor (TCR) signaling via alteration of expression of genes such as *UBASH3A*, *CD28* and *CSK*^30–32^. Overall, these observations confirm the importance of CD4+ T cell activation in CeD but do not delineate how CeD-associated SNPs affect gsTcells upon activation.

This knowledge gap is partially due to an incomplete understanding of the regulation of the response to stimulation in gsTcells. Recently, it was shown that the genetic risk loci associated with CeD are enriched with binding sites of specific transcription factors (TFs), including STAT4, STAT5A, STAT5B, T-BET, AP-1 subunit FOS and TFs from the NFκB signaling pathway^29^. Indeed, many of these TFs have been implicated in regulation of CD4+ T cell activation or in CeD^12,33–36^. However, the role of these TFs, as well as the dynamic transcriptional and epigenetic response in the activation of gsTcells, has not been described. Nor has the role of CeD-associated genetic variants in these dynamic transcriptional processes been explored in gsTcells.

Here, we set out to profile the transcriptomic and epigenetic response of gsTcells derived from CeD patients upon TCR-stimulation with anti-CD3 and anti-CD28 (aCD3/aCD28). This allowed us to identify the regulatory steps essential for the rapid and robust activation of cytokines important for CeD etiology and to prioritize which CeD-associated risk loci are related to the activation of gsTcells. Overall, we elucidate the dynamic events in gsTcells that can be induced by gluten peptides in CeD patients.

## Results

### Dynamic transcriptome changes in stimulated gsTcells

**Figure 1:**
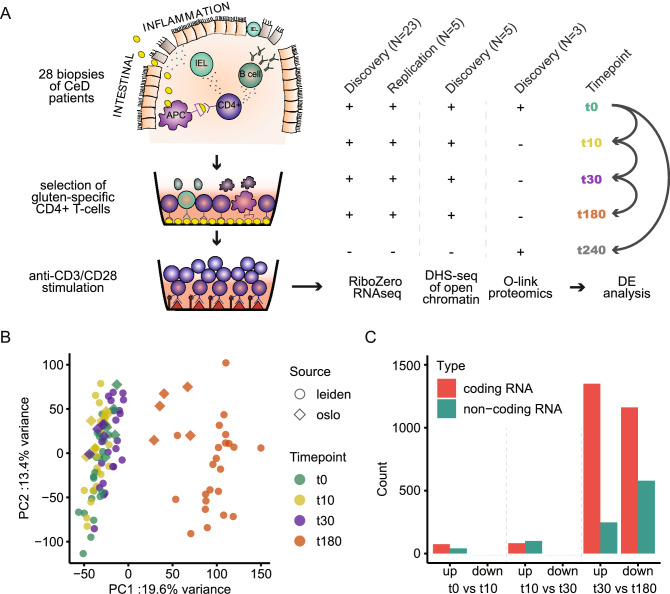
Stimulation of gluten-specific CD4+ T cells with aCD3/aCD28. (**A**) Experimental scheme of the discovery and replication cohort. The ‘+’ symbol indicates that a measurement is available at that timepoint. 28 gsTcell clones were isolated from CeD biopsies, 23 clones were used in discovery analysis and 5 were used for replication and DHS-sequencing. A final 3 clones from the discovery set were used for proteomic analysis. (**B**) PCA of the complete expression data of the discovery (circles) and replication (diamonds) cohorts. Each time point is indicated in a different color. (**C**) Differentially expressed genes identified by differential expression (DE) analysis between consecutive timepoints, plotted per comparison. Biotypes and direction of each DE gene are indicated.

To study the TCR-induced response of gsTcells, we opted for aCD3/aCD28 stimulation as a proxy for the interaction of gsTcell-TCR with gluten peptides presented by antigen-presenting cells in the context of HLA-DQ2 or -DQ8. Twenty-three CD4+ gsTcell clones isolated from biopsies from patients with active CeD were cultured and stimulated in vitro and used to perform transcriptomic (RNA-Seq; n = 23) and targeted proteomics analysis (n = 3) (discovery cohort, Suppl. Table 1). An additional five gsTcell clones were used for replication of the transcriptomic data and detection of open chromatin through DNase-Hyper-Sensitivity sequencing (DHS-sequencing) (Fig. 1A, Suppl. Table 1). The transcriptomic response of gsTcells to stimulation showed strong and consistent effects after 180 min relative to the earlier timepoints. Although there was considerable inter-clonal variation, the replication and discovery cohorts behaved very similarly (Fig. 1B). We used the discovery cohort to determine the dynamic transcriptional response of genes during the course of the stimulation, which showed clear distinctions between each time point (Suppl. Fig. 1). We performed differential expression (DE) analysis between consecutive timepoints to reveal the changes in gene expression over time. Between 0‒10 min, 10‒30 min and 30‒180 min, 115, 182 and 3339 DE genes were identified, respectively (Fig. 1C, Suppl. Table 2). Finally, non-coding genes were found to be differentially expressed at all timepoints, but at 180 min the downregulated set was roughly twice as large as the upregulated set, which was not the case for the coding genes (Fig. 1C). GsTcell clones thus rapidly displayed strong and dynamic transcriptional changes after stimulation.

### DE genes cluster into response patterns with distinct functions

**Figure 2:**
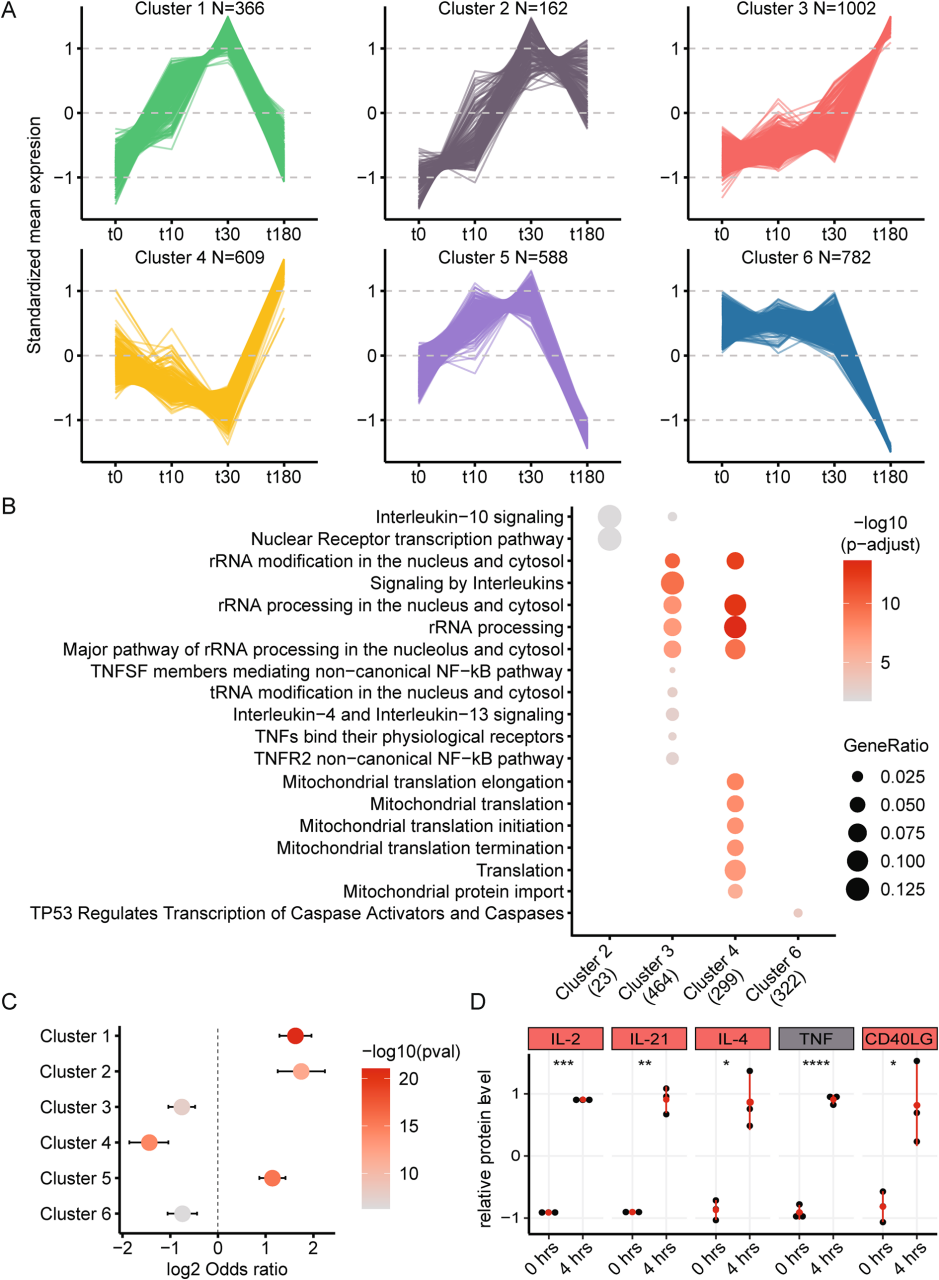
Differentially expressed genes cluster into response patterns showing distinct functions. (**A**) Cluster analysis identified 6 robust clusters encompassing the response profiles of all 3509 differentially expressed (DE) genes. Numbers of genes per cluster are shown. Y-axis shows the mean expression of the genes at each respective time point, centered to mean zero and scaled to standard deviation one. (**B**) Reactome gene set enrichment analysis shows enriched pathways for 4 out of the 6 clusters. (**C**) Enrichment analysis by Fisher’s exact test of non-coding RNAs per cluster. Significance is shown in shades of red. Log2 odds ratios are plotted on the x-axis and indicate enrichment or depletion. Error bars indicate 0.95 confidence intervals. (**D**) Scaled relative protein levels (Olink) in the unstimulated condition and after 4 h of stimulation for the three independent experiments (black dots). Box colors above the dotplots indicate the cluster in which the DE genes are found. Mean, minimum and maximum relative protein levels are indicated in red. Nominal significance is indicated by asterisks (*p value < 0.05, **p value < 0.01, ***p value < 0.001 and ****p value < 0.0001).

To understand and categorize the activation of gsTcells, we clustered the DE genes using k-means clustering to identify temporal response patterns. This identified six distinct clusters that each represent a specific response (Fig. 2A, Suppl. Fig. 2, Suppl. Table 2). Genes in Clusters 1 (n = 366) and 2 (n = 162) were upregulated early (at 10 min). In contrast, cluster 3 genes (n = 1002) were upregulated after 30 and 180 min. Cluster 4 (n = 609) genes displayed an early decrease but recovered after 180 min. Cluster 5 (n = 588) genes responded similarly to cluster 1 genes, but their gene expression levels after 180 min were decreased compared to unstimulated expression levels. Finally, cluster 6 genes (n = 782) show a consistent late decrease in expression.

Gene set enrichment analysis using the Reactome database pinpointed enriched gene sets (false discovery rate (FDR) < 0.05) for clusters 2, 3, 4 and 6 (Fig. 2B) but found no enrichment in clusters 1 and 5. Cluster 2 is enriched for Nuclear Receptor transcription pathway genes (*NR4A1*, *NR4A2* and *NR4A3*) and IL10 signaling genes (*TNF* and *ICAM1*). Cluster 3 is predominantly associated with immune function, showing an enrichment of cytokine signaling pathway genes. Moreover, genes associated with transcriptional and translational processes are also enriched in cluster 3, consistent with an immune response that requires the production and secretion of cytokines and other signaling proteins. Cluster 4 is enriched in translational and mitochondrial response genes that are associated with a shift towards protein production. Finally, the downregulated genes in cluster 6 are enriched in genes involved p53-mediated regulation of caspases. Downregulation of cluster 6 genes may thus decrease apoptosis and cell death and favor proliferation, paving the way for a robust immune response by gsTcells that is mediated by the genes in cluster 3.

To ascertain how these pathways might be regulated, we investigated the relative enrichment of non-coding RNAs (ncRNAs) per cluster. The early-responding clusters 1, 2 and 5 are enriched for ncRNAs, implying that these RNAs play a role in regulating expression of genes at 180 min, when protein-coding genes are enriched (clusters 3, 4 and 6) (Fig. 2C). Several genes encoding TFs that mediate early immune and stress responses are also found in clusters 1 and 2. These TFs include all *EGR* TFs, *NR4A1*, *NR4A2*, *NR4A3*, *ATF3, FOS* and *FOSB*, of which the latter two are subunits of AP-1 (Suppl. Fig. 3A). Additionally, cluster 3 encompasses *REL* (encoding NFκB subunit c-REL), *NFκB1* (encoding NFκB subunit p50) and the NFκB inhibitory genes *NFκBIA*, *NFκBID* and *NFKIZ*, which suggests an early activation of the NFκB pathway and a subsequent feedback loop after 180 min (Suppl. Fig. 3B)^37,38^. Thus, the action of several TFs that are either activated or transcribed soon after stimulation, possibly in conjunction with ncRNAs, seems to mediate the strong response of genes after 180 min.

**Figure 3:**
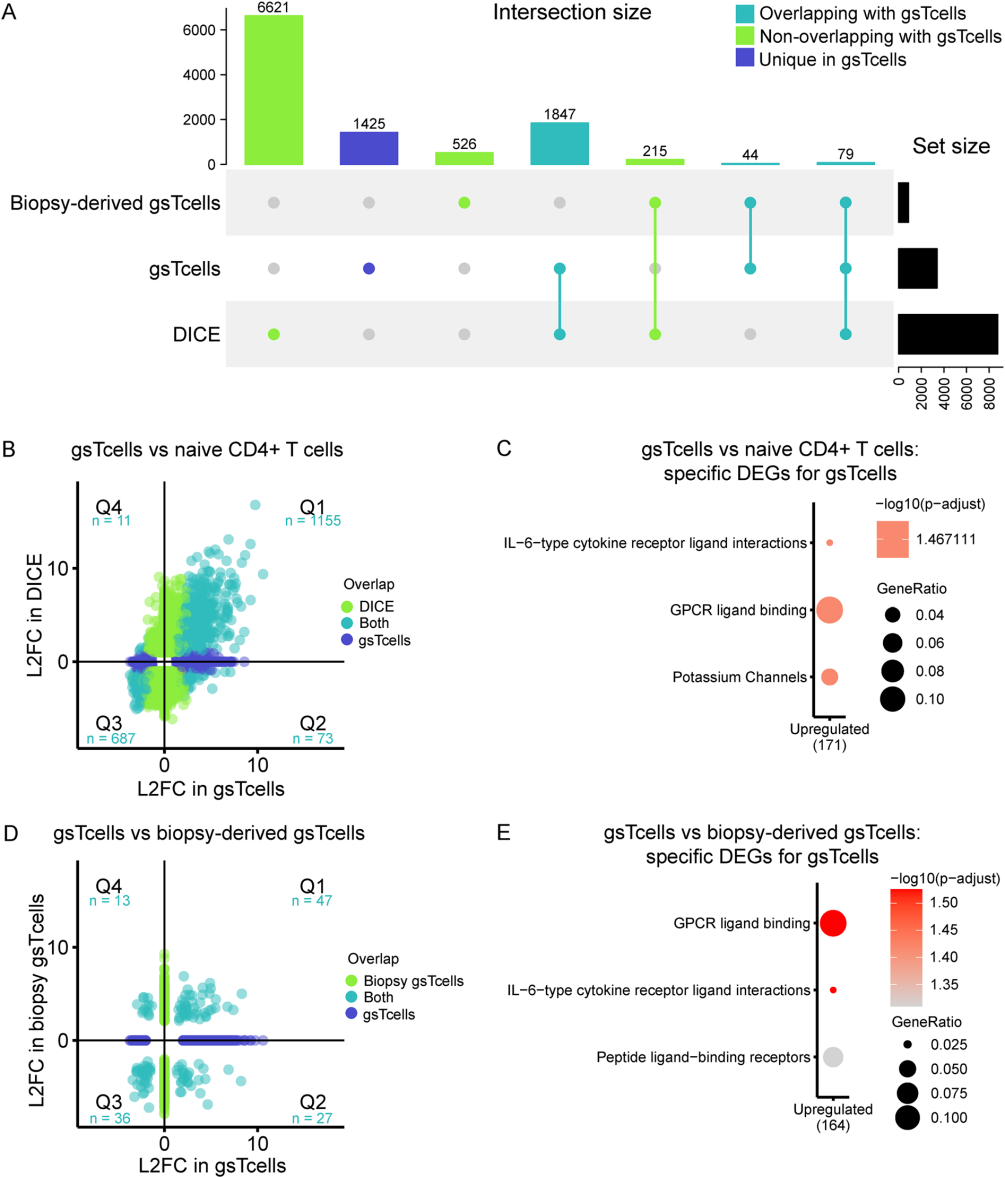
The transcriptional response of gsTcells shows differences and similarities with other T cells. (**A**) Upset plot comparing significant DE genes between stimulated naïve CD4+ T cells (DICE) (t0 vs t240, FDR < 0.05 and absolute log2FC > 1)^41^, gsTcells (t0 vs t180, FDR < 0.05 and absolute log2FC > 1) and biopsy-derived gsTcells (gsTcells vs CD4+ T cells in cases/controls, FDR < 0.05 and absolute log2FC > 2)^13^. At the top, the size of the intersecting sets with gsTcells are indicated in light blue, non-overlapping genes with gsTcells are shown in light green and genes unique to gsTcells are shown in dark blue. The right barplot shows the total number of DE genes per dataset. (**B**) Scatterplot of log2FC of DE genes between DICE (y-axis) and gsTcells (x-axis). Numbers in light blue indicate the number of genes in each quadrant that are significant in both analyses. (**C**) Gene set enrichment analysis done using Reactome of DE genes unique for gsTcells as compared to DICE naïve CD4+ T cells (adjusted *p* value < 0.05, absolute log2FC > 2). At the bottom is the direction of expression of the DE genes in gsTcells. Numbers in brackets indicate the number of DE genes present in all enriched pathways. Dot size indicates the ratio of the number of genes present in the gene set and the total gene set used in each pathway. (**D**) Comparison as in (**B**) for DE effects in CeD biopsy-derived gsTcells (y-axis). (**E**) Gene set enrichment analysis as in (**C**) for genes unique to gsTcells compared to CeD biopsy-derived gsTcells and DICE naïve CD4+ T cells (adjusted *p* value < 0.05, absolute log2FC > 2).

To confirm that transcriptional changes lead to secretion of cytokines, we measured a panel of 92 proteins in the gsTcell culture medium after 4 h of stimulation. Thirty of these proteins are encoded by genes differentially expressed during the stimulation of gsTcells (Suppl. Fig. 4A, Suppl. Table 3). We found that levels of IL-21, CD40LG, IL-2, IL-4 and TNF were significantly increased (nominal *p* value < 0.05) (Fig. 2D) in concert with increased expression of their corresponding genes (Suppl. Fig. 4B). These cytokines all have pro-inflammatory roles and contribute to activation and proliferation of other cell types, including B cells (IL-21, IL-4 and CD40LG) and other T cells like IELs (IL-2 and TNF)^39,40^.

**Figure 4:**
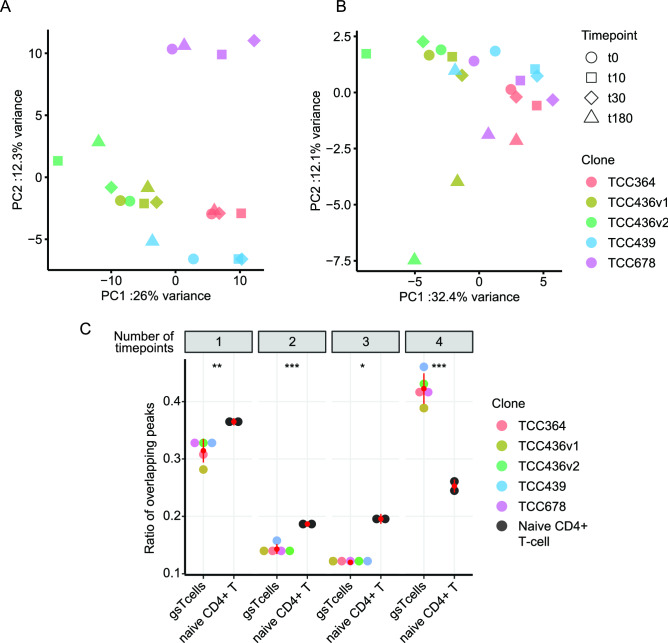
Open chromatin changes are minimal in gsTcells upon stimulation. (**A**) PC analysis of all peaks in each individual gsTcell clone in the discovery cohort. Timepoints indicated by shapes, clones by colors. (**B**) As in (**A**), but only using peaks within a 5 KB window around the transcription start sites of the 3509 DE genes in the PCA. (**C**) Comparison of the relative changes in the individual gsTcells and naïve CD4+ T cells of two individuals between all timepoints. Overlaps between all timepoints were calculated using the multiinter Bedtools function, and the relative number of overlaps is plotted for each. Peaks that are present at all timepoints are represented in the ‘4’ category. Peaks unique to one time point are represented in the ‘1’ category. Clones are indicated with the same colors as in (**A**) and (**B**). Nominal significance indicated with asterisks (*p value < 0.05, **p value < 0.01 and ***p value < 0.001).

In summary, the distinct and dynamic transcriptional changes we observe represent a robust translational immune response to TCR activation that leads to the secretion of several cytokines within 4 h.

### Transcriptional changes identified in gsTcells upon activation are similar to those in other T cells

To examine the specificity of the gsTcell response, we compared the DE genes of gsTcells (between 0 and 180 min) to those of naïve CD4+ T cells stimulated with aCD3/aCD28 [DICE consortium (n = 90)]^41^. A large proportion (57%) of the DE effects we observed in gsTcells were also found in the DICE consortium data (96% directional concordance; Fig. 3A, Suppl. Tables 4, 5). Directionally concordant genes (Q1 and Q3 in Fig. 3B) showed an enrichment for genes involved in interleukin signaling and rRNA processing (Suppl. Fig. 5A), as we had observed in cluster 3 (Fig. 2B), suggesting this to be a general response of CD4+ T cells to stimulation. Genes uniquely activated in gsTcells include chemokines such as *CCL1*, *CXCL1* and *CCL4L1,* which encode for peptides that bind the receptors CCR8, CXCR2 and CCR5 and that can mediate recruitment of immune cells, including Type 2 innate lymphocyte cells, neutrophils and activated CD8+ T cells, respectively (Fig. 3C, Suppl. Fig. 6A)^42^. In addition, the cytokine-encoding genes *IL5*, *IL9*, *IL19, IL17F* and *IL26,* as well as *RORC (*encoding for TF RORγt*)*, are uniquely differentially expressed in gsTcells, albeit at low levels, and each points to a different subset classification of T helper cells for gsTcells^43^.

**Figure 5:**
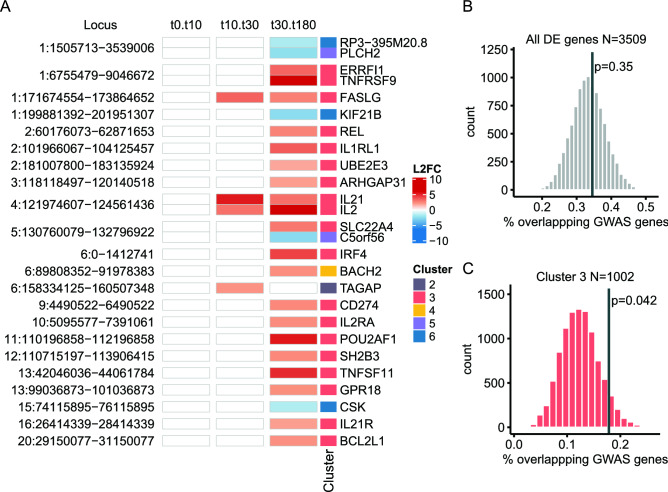
DE genes show enrichment for CeD loci. (**A**) Genes prioritized from CeD loci^30^ were overlapped with DE genes and plotted per CeD locus. Log2 fold change between timepoints is indicated, and clusters are depicted with colored boxes (right). (**B**) Enrichment of DE genes over the null distribution (histogram) in CeD loci (± 125 kb window around start and end of gene) using GREA^19^ for all DE genes (n = 3509). X-axis indicates the number of genes that overlap with CeD loci as a percentage. The histogram shows the null distribution based on 10,000 permuted gene-sets. The black line indicates the value of the true gene-set. Nominal *p* values are indicated. (**C**) As in (**B**), but only for genes in cluster 3 (n = 1002).

**Figure 6:**
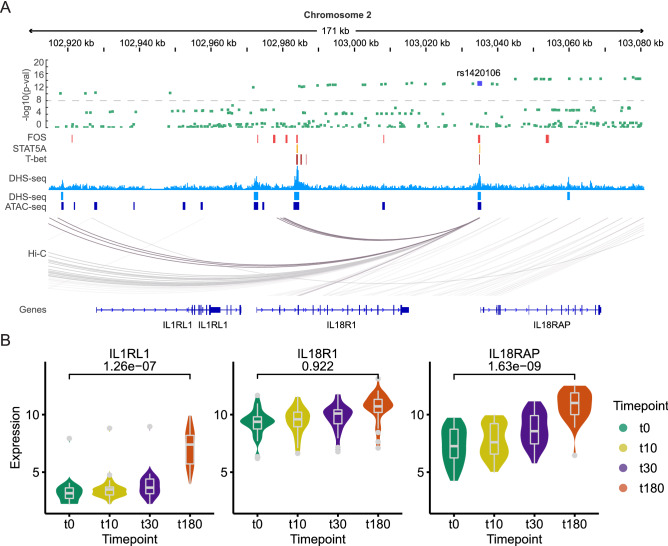
Rs1420106 in the *IL1RL1/IL18R1/IL18RAP* locus overlaps with open chromatin and TF binding sites in gsTcells. (**A**) Overview of the *IL1RL1/IL18R1/IL18RAP* locus. From top to bottom: summary statistics of the CeD GWAS meta-analysis^22^ (green); TF binding sites based on Chip-seq of *FOS* (red), *STAT5A* (orange) and *T-BET/TBX21*^29^ (brown); DHS profile of unstimulated gsTcells with peak calls depicted below (light blue); peaks from naïve CD4+ T cell ATAC-seq^46^ (dark blue); capture Hi-C data of activated CD4+ T cells depicting the 3D interactions between the highlighted region and other locations in the locus^82^ (grey) and the gene annotations. The prioritized SNP rs1420106 is indicated in dark blue. (**B**) Gene expression pattern of genes in the locus. Y-axis represents the VST-normalized expression data. Black line and number indicate the adjusted *p* value (DeSeq2) of the DE effect between the t0 and t180 timepoints. Blood eQTL *p* values of SNP rs1420106 for the indicated genes are: *IL18RAP*, 3.27e−310; *IL1RL1*, 1.95e−144 and *IL18R1*, 1.63e−185 (eQTLgen.org^50^).

To determine which T helper subset the gsTcells most resemble, we compared the expression patterns of the main cytokines associated with Th1 (*IFNG*, *TNF*), Th2 (*IL4*, *IL5*, *IL13*), Th17 (*IL17A*), Treg (*IL10*, *TGFB1*), T follicular helper (*IL21*) and Th9 (*IL9*) cells (Suppl. Fig. 6B)^6–13,43,44^. We observed that cytokines characteristic for several subsets are strongly upregulated upon stimulation, with Th1 cytokines most strongly expressed. GsTcells are therefore most similar to Th1 cells but also share characteristics with multiple Th subsets.

Overall, the strong concordance between the TCR-induced response profile of naïve CD4+ T cells and gsTcells (Fig. 3A, B) suggests that the TCR response is largely shared in CD4+ T cells. However, we also observed 1371 unique DE genes that were not observed in the naïve CD4+ T cells or biopsy-derived gsTcells. This set of genes was mainly upregulated (1047/1371) and was enriched for G protein‒coupled receptor ligand binding, IL-6–type cytokine receptor ligand interaction and peptide ligand‒binding receptors (Fig. 3E). Nonetheless, when compared to naïve CD4+ T cells, in vitro*-*cultured gsTcells show a distinct response profile on top of the shared TCR response profile that includes a diverse set of cytokines and chemokines that are comparable to that of Th1, Tfh and Th2 cells, which is consistent with previous evidence^6–13,43,44^.

### The expression profile of in vitro*‒*cultured gsTcells is similar but not identical to the expression profile of gsTcells isolated directly from biopsies

Next, we compared the transcriptomic response of our cultured gsTcells with gsTcells taken directly from CeD biopsies 6 days after an in vivo gluten challenge^13^. These biopsy-derived gsTcells were obtained from Christophersen et al.^13^ and contained tetramer+ CD4+ T cells and tetramer− CD4+ T cells from CeD patients (n = 5) and gut CD4+ T cells from healthy controls (n = 4). We found that 123 out of 3395 DE genes in gsTcells are shared with the 865 DE genes from biopsy-derived gsTcells (*p* value = 0.0056; two-sided Fisher’s exact test) (Fig. 3D, Suppl. Tables 4, 6). These overlapping genes were enriched for the 'TNFR2 non-canonical NFκB pathway’ in Reactome and included immune genes such as *CD200*, *MAP4K3* & *PDCD1*, *IL21* (a key regulator in CeD) and *IL22* (a regulator of intestinal epithelial homeostasis^45^) (Suppl. Fig. 5B, Suppl. Table 7). The differences between the in vitro-cultured gsTcells and biopsy-derived gsTcells could partly be attributable to differences in sample size. Moreover, the in vitro‒cultured gsTcells were stimulated for 3 h, and the DE genes therefore reflect early transcriptional changes upon stimulation, which may not reflect the activation state of in vivo biopsy-derived gsTcells. Finally, continuous in vitro culturing of the gsTcell clones in the presence of IL-2 and IL-15 is likely to introduce changes in the expression profile of the gsTcell clones compared to the biopsy-derived gsTcells. Thus, gsTcells show disease-relevant characteristics but are not identical to ‘fresh’ biopsy-derived gsTcells.

### Chromatin of gsTcells remains largely stable

Next, we assessed if changes in chromatin state agreed with changes in gene expression upon stimulation of gsTcells. We observed that the chromatin state measured by DHS-seq remained mostly stable at all four timepoints when assessed genome-wide, and most of the variation in open chromatin was explained by which gsTcell clone the data was derived from, and not by stimulation effects (Fig. 4A, Suppl. Table 8). Nonetheless, when investigating the open chromatin sites in a 5 kb window around the 3509 DE genes, we observed some variation between t180 and the other timepoints (Fig. 4B). However, differential peak-calling analysis did not find any sites with a log2 fold change > 1. These results are probably due to the large variation between the clones (Fig. 4A, B) and the modest sample size. We also investigated the overlap between peaks in each clone and compared that to the overlap between ATAC-seq peaks in naïve CD4+ T cells in a similar stimulation experiment^46^. Overall, the naïve CD4+ T cells showed more stimulation-specific effects, with 25% of peaks being shared at all four timepoints, as opposed to gsTcells, where 45% of the peaks are shared (Fig. 4C). Moreover, TF binding site enrichment analysis on peaks uniquely present in unstimulated gsTcells, but not seen in naïve CD4+ T cells under unstimulated conditions, showed that AP-1 binding sites are enriched in gsTcells even prior to stimulation (HOMER, *p* value = 1e−16). Altogether, this suggests that the early upregulation of gene expression upon activation in gsTcells is mostly independent of chromatin state and instead driven by the interplay of TFs and ncRNAs.

### DE genes show enrichment for CeD loci and CeD-associated genes

To ascertain if the DE genes we found are affected by the genetic background associated with CeD, we integrated our transcriptional data with 118 *cis*-genes for CeD identified by a previous gene prioritization effort^30^ (Fig. 5A). We observed that 26 of the prioritized genes are DE in the gsTcells. Of these 26 genes, 18 belong to cluster 3, in which immune response pathways are overrepresented, consistent with the mostly T cell–based GWAS signal of CeD^23^. Of particular interest are *IL21* and other cytokine-encoding genes (*IL2*, *TNFSF11* and *FASLG*), several cytokine receptor genes (*IL21R*, *IL1RL1*, *TNFRSF9* and *IL2RA*) and genes for TFs that can play a role in immune response (*REL*, *BACH2* and *IRF4*). Interestingly, *UBE2E3*, *CSK* and *SLC22A4* are DE in gsTcells but not in naïve CD4+ T cells (Fig. 3B), which implies that these genes may have a relatively specialized function in gsTcells.

We subsequently checked if the DE genes were overrepresented in CeD loci^23^ using genomic region enrichment analysis (GREA)^19^ (Fig. 5 B,C; Suppl. Fig. 8). GREA operates by comparing the overlap of DE genes in a window of ± 125 kb around a CeD locus to the overlap found randomly in a permutation-based null distribution. We found no significant enrichment for genes located in CeD loci when assessing all 3509 DE genes (Fig. 5B). However, when we assessed the enrichment per cluster (Suppl. Fig. 8), cluster 3 showed a nominally significant enrichment for genes located in CeD loci (*p* value = 0.042; Fig. 5C), with around 18% of cluster 3 genes being located near a CeD locus. Thus, the genes in cluster 3 may be affected by the genetic predisposition for CeD.

Next, we assessed whether any of the TFs that were DE in gsTcells were enriched to bind in CeD loci using REgulatory trait Locus Intersection (RELI) in conjunction with the provided database of ChIP-seq data for 389 TF‒cell type pairs^29^. In total, 98 TF–cell type pairs showed significant enrichment for binding in CeD loci (FDR < 0.05) (Suppl. Table 9). Of these TFs, FOS, STAT5A and T-BET (encoded by the gene *TBX21*) were of particular interest as their genes showed a DE effect in gsTcells and the ChIP-seq data used in RELI was derived from CD4+ T cells. *FOS* falls in cluster 1 and showed an early but transient response to stimulation (Suppl. Fig. 9A), whereas *STAT5A* and *TBX21* showed a later response corresponding to the cluster 3 profile (Suppl. Fig. 9B and C, respectively). FOS is a well-known early response immune and stress response TF that can heterodimerize with the JUN or ATF TF families. Indeed, *ATF3* is also DE and has a very similar transcriptional profile to *FOS* (Suppl. Fig. 3A). STAT5A is required for IL2 signaling in CD4+ T cells^47^, and T-BET is the major TF for Th1 differentiation, expression of IFNγ and other Th1-specific cytokines^48^. Thus, these TFs all have prominent roles in CD4+ T cell differentiation and activation.

To explore if any of the SNPs in the 43 CeD loci from the latest CeD meta-analysis^23^ could directly affect gene regulation in gsTcells, we overlapped the TF binding sites of FOS, STAT5A and T-BET with the open chromatin regions, CeD GWAS summary statistics^32^, DE information, ATAC-seq of naïve CD4+ T cells^46^ and capture Hi-C data of activated CD4+ T cells (Fig. 6). We found one locus of interest near the *IL18RAP/IL1RL1* genes. In this locus, one SNP (rs1420106, GWAS *p* value 8.7e−14) located in the promoter of *IL18RAP* overlapped with all three of the enriched TF binding sites, a DHS peak from gsTcells and an ATAC-seq peak from naïve CD4+ T cells (Fig. 6A). We assessed if this locus could act as an enhancer using publicly available capture Hi-C data from activated CD4+ T cells^49^ and found interactions with the promoters of *IL1RL1* and *IL18R1* (Fig. 6A). Moreover, rs1420106 strongly affected the expression of *IL18RAP*, *IL1RL1* and *IL18R1* (eQTL *p* values 3.27e−310, 1.95e−144 and 1.63e−185, respectively) in whole blood (eQTLgen database^50^). Finally, both *IL1RL1* and *IL18RAP* were significantly upregulated in gsTcells at t180 compared to t0 (Fig. 6B). Together, this suggests that rs1420106 might have a role in the activation response of gsTcells by modifying the expression of *IL18RAP*, *IL1RL1* and *IL18R1*. Similarly, we identified three more CeD loci (containing genes *BACH2*, *IL21*-*IL2* and *TAGAP*) that showed overlap of SNPs associated with CeD with DHS sites found in gsTcells that also bind the TFs FOS, STAT5A and/or T-BET (Suppl. Figs. 10–12).

Thus, CeD-associated genetics may play a complex role in gsTcells during activation of these important cells in CeD pathology.

## Discussion

In this study, we characterized gsTcells, one of the key players in CeD pathogenesis, by profiling transcriptomic and epigenetic changes during the early response to aCD3/aCD28 activation. We pinpointed pathways and TFs that may regulate these cells and confirmed that gsTcells are not restricted to any specific class of Th cells, but rather express cytokines, chemokines and TFs that are characteristic of Th1 but also of Tfh and Th2 subsets. We also identified *CCL1, CXCL1* and *CCL4L1* as unique DE genes that had not previously been shown to be expressed by CD4+ T cells in the context of CeD. Finally, we showed that the early response of gsTcells to stimulation is regulated by rapid upregulation of many TFs and ncRNAs in combination with the activation of JNK/AP-1 and NFκB TFs, whereas changes in chromatin were minor. Overall, our results provide an in-depth analysis of the molecular pathways that are activated in gsTcells upon TCR activation.

Our study illustrates that gsTcells express and secrete cytokines that can be associated to various subsets of Th cells, most prominently with the Th1 (*IFNG* and *TNF*), Th2 (*IL4*, *IL5*, *IL13*) and Tfh (*IL21*) subsets, which is in agreement with previous studies^6–13,43,44^. Based on their unique cytokine profile, gsTcells may exert multiple functions in CeD pathogenesis. Firstly, IFNγ is important for eliciting a strong response to foreign antigens such as the gluten peptides in CeD^51^. Moreover, IFNγ can also directly affect the integrity of the intestinal barrier^52^. Secondly, Th2-associated cytokines and IL-21 produced by Tfh cells are important for plasma cell differentiation, B cell activation and autoantibody production^43^. Finally, IL-21, IL-2 and TNF have been shown to activate CD8+ T cells and IELs in the gut, which thereby become “licensed to kill” epithelial cells, leading to the villus atrophy in CeD^12,19,20,53^. Thus, cytokines derived from gsTcells may play a role in several distinct disease mechanisms.

Our comprehensive analysis of the regulatory mechanisms that drive gene expression in activation of gsTcells agrees in part with the results of a study where the authors analyzed biopsy-derived gsTcells (Christophersen et al.^13^) but also uncovered differences. These differences might be due to differences in sample size and experimental design between the two studies but may also reflect the fact that the gsTcells used in our study have been expanded in vitro in the presence of IL-2 and IL-15. Nonetheless, the unique expression of genes that are not DE in activated naïve CD4+ T cells and the enrichment of genes that overlap with genes specific to biopsy-derived gsTcells suggest that in vitro*‒*cultured gsTcells are a unique and appropriate model to delineate the dynamic transcription and regulation of gsTcells.

Similarly, comparison with other datasets such as the DICE data (Fig. 3A, B) and CeD-patient derived CD4+ T cells from PBMCs described by Quinn et al. (data not shown), validate our findings that the gsTcells have a similar but distinct expression profile and that important cytokines and other genes are associated to CeD etiology, such as *IFNG, IL21, IL17A* and *IL4*^54^, are differentially expressed. Still, the unique expression profile of gsTcells relative to naïve or blood-derived CD4+ T cells may be lost if the comparison with other antigen-specific T cells or memory CD4+ T cells activated with similar stimuli and timepoints.

Based on the expression pattern of the DE genes, we identified six major gene clusters with distinct dynamic responses and functions. Early responding genes were represented by clusters 1, 2 and 5 and contained multiple TFs, some cytokines and a disproportionate number of ncRNAs, implying that these genes regulate the response at later time points. An earlier study also observed a ncRNA response during the activation of lymphocytes, which supports the idea that ncRNAs are key in the development and activation of CD4+ T cell^55^. Some ncRNAs that are DE in gsTcells have also been implicated in immune activation, inflammation and proliferation in other studies, including *LINC00174*, *AF131217.1*, *TINCR* and *LINC00342,* which were all found in cluster 1^56–60^. Thus, ncRNAs seem to play a pivotal role in the immune response by changing the expression of specific genes in gsTcells.

The gsTcells we studied showed a stable open chromatin profile genome-wide, with only minor changes upon stimulation near the transcription start sites of DE genes. However, we cannot exclude the possibility that the stability we observe is a consequence of culturing gsTcells in the presence of cytokines to induce expansion^61^. In contrast to the stable open chromatin profiles in gsTcells, the transient expression of specific TFs and ncRNAs, in concert with the activation of common signaling pathways like JNK/AP-1 and NFκB, may be the source of the unique expression profile observed in the gsTcells.

Several DE genes are located in CeD-associated loci, and we found a subtle enrichment for genes of immune cluster 3 in CeD loci. However, the largest CeD association study to date was performed using the Immunochip platform, which is enriched for known immune regions, and thus we may have missed enrichment in other functional clusters. Nonetheless, 18% of the genes in cluster 3 are located in CeD loci, highlighting that TCR-mediated T cell activation, particularly in gsTcells, may be affected by CeD-associated SNPs.

To ascertain the role of CeD-associated SNPs in regulating gene expression in gsTcells, we integrated multiple publicly available functional data layers with the gene expression and DHS regions of the gsTcells and CeD-associated SNPs. While this integration provides suggestive evidence that the prioritized SNPs have regulatory potential in gsTcells, we cannot directly confirm that the genetic effect has a regulatory role. This would require an eQTL analysis with primary gsTcells derived from biopsies or functional validation by targeting these candidate regulatory elements, both of which are beyond the scope of this study. Despite these challenges, we provide evidence for potential genetic interference of CeD-associated SNPs in gsTcells in several loci and pinpoint several SNPs and regulatory regions within the genome that are the most likely candidates to cause this interference.

In summary, we present an in-depth characterization of early transcriptional dynamics of gsTcells in response to TCR activation. We highlight that this transcriptional response is most likely regulated by TFs and ncRNAs rather than large changes in chromatin state. Finally, we prioritize several CeD-associated genetic loci that may impact the TCR-activation in gsTcells directly.

## Methods

### Obtaining T cell clones from biopsies of CeD patients

Gluten-specific CD4+ T cells were isolated from CeD patient small intestinal biopsies, as described previously^25,62–65^. Patients were diagnosed with CeD with small bowel biopsy confirmation and included at Leiden University Medical Center (LUMC), the Netherlands (n = 18), and the Riks Hospital in Oslo (RHO), Norway (n = 4), from which 23 and 5 gluten-specific T cell lines were isolated, respectively (Suppl. Table 1). Briefly, written informed consent was given by all patients and the small intestine biopsies from CeD patients were cultured with a mixture of gluten and TG2-treated gluten (deamidation) for 5 days. To expand the T cells, IL-2 (20 Cetus units/ml; Novartis, Arnhem, the Netherlands) and IL-15 (10 ng/ml; R&D systems, Abingdon, UK) were added. Subsequently, irradiated allogeneic peripheral blood mononuclear cells in the presence of phytohemagglutinin (1 μg/ml; Remel Inc. Lenexa, USA), IL-2 (20 Cetus units/ml) and IL-15 (10 ng/ml) were mixed with T cells for re-stimulation^25^. The resulting T cell clones were tested for reactivity against gluten digested by pepsin and trypsin and TG2-treated in proliferation assay. The pepsin/trypsin digest of gluten was prepared as described by Van de Wal et al.^63^. For deamidation, the pepsin/trypsin-digested gluten (500 mg/ml) was incubated with 100 mg/ml of guinea pig tTG (T-5398; Sigma, St. Louis, MO) at 37 °C for 2 h in PBS with 1 mM CaCl_2_ and subsequently used in T cell proliferation assays. Proliferation assays were conducted as described by Van de Wal et al.^63^, for samples from LUMC, and Molberg et al.^65^, for samples from RHO. Gluten-specific lines were cloned by limiting dilution and expanded again by re-stimulation at 1- to 3-week intervals^25^. Clones were stored in liquid nitrogen. All methods were performed in accordance with relevant guidelines and regulations.

### Stimulation of T cell clones

In all, 28 gluten-specific T cell clones were stimulated in 6-well plates coated overnight with anti-CD3 (2.5 μg/ml; Biolegend, San Diego, CA, USA) and anti-CD28 (2.5 μg/ml; Biolegend) or PBS (negative control) for 0, 10, 30 and 180 min. At each timepoint, cells were harvested for RNA isolation. Cell culture medium was harvested after 240 min for proteomic analysis.

### RNA isolation and library preparation of stimulated gsTcells

GsTcells were harvested at each time point, washed with PBS and resuspended in a lysis buffer (Ambion, Life Technologies, Carlsbad, CA, USA). RNA was extracted with the mirVana RNA isolation kit (Ambion) according to the manufacturer’s instructions. The quantity and quality of RNA was determined by Bioanalyzer (Agilent technologies, Santa Clara, CA, USA). The sequencing libraries were prepared from 1 μg of total RNA using the TruSeq Stranded Total RNA with Ribo-Zero Globin kit (Illumina, San Diego, CA, USA) according to the manufacturer’s instructions. Sequencing was done with the Illumina HiSeq2500 (Illumina).

### DNase I hypersensitivity sequencing and analysis

Standard protocols for nuclei isolation, DNase I (Roche #04716728001) treatment and library preparation for DNase I hypersensitivity sequencing generated within the Blueprint consortium were followed. Protocol details^66^ can be found at: http://www.blueprint-epigenome.eu/UserFiles/file/Protocols/Blueprint_DNase1_Protocol.pdf. All samples were sequenced to a sequencing depth of approximately 50–60 million 50 bp single-end reads.

### Protein analysis

Supernatants from unstimulated and 4 h-stimulated gsTcell cell cultures were taken and analyzed with the Immuno-Oncology panel of Olink (http://www.olink.com/products/immuno-oncology). Data was analyzed by subtracting relative log2 protein levels in a blank medium control from the relative log2 protein levels in the supernatants, followed by a two-sided *t*-test to measure significant change between supernatants of unstimulated and 4 h-stimulated gsTcells.

### Statistical methods

Statistical analyses were performed in R (version 3.6.3)^67^ unless otherwise specified. Visualization of results was done using the R package ggplot2 (version 3.3.0)^68^.

### RNA-seq quantification

Before alignment, the reverse complement of the fastQ sequences were taken using the FASTX-Toolkit^69^. Alignment was done using Hisat2 (version 2.0.4)^70^ against the forward strand, with default alignment parameters. The reference genome index was made using the Hisat2-build indexer and 1000 genomes reference genome version GRCh37 v75 with default parameters. For the samples that had paired-end data, only the first mate file was used for alignment. Reads mapping to multiple positions were removed. The genes were quantified using HTSeq (version 0.6.1.p1)^71^ with options -m union, -t exon, -stranded yes and other options on default.

### DE analysis

The raw count matrix, containing 63,682 genes and 112 samples (92 samples from the Leiden cohort and 20 from the Oslo cohort), was first filtered to remove any non-expressed genes by selecting only genes that had at least 1 read in 20 samples. This resulted in 29,772 genes to be tested for DE effects. Samples from the Leiden and Oslo cohorts were then split, and the DE effects assessed separately.

DE effects were quantified using the R package DEseq2 (version 1.26.0)^72^, including RNAseq batch and sex as covariates for the Leiden samples. No covariates were included for the Oslo samples because no sex information was available and all samples had been sequenced in the same batch. DE effects were then mapped between the t0 and t10, t10 and t30, and t30 and t180 timepoints. DE effects in the Leiden cohort were filtered on having an absolute log2 fold change (log2FC) of at least 1 and an FDR < 0.05. Oslo samples were used as the replication cohort, and comparisons between the two were made using unfiltered Oslo data. In total, we identified 3509 unique DE genes in the Leiden cohort. These genes were used for interpretation and downstream analysis. PC analyses were performed on the variance-stabilized count data.

### Clustering of DE genes into distinct response patterns

DE genes were clustered into time patterns as follows. The gene expression matrix was VST-normalized using DESeq2 (version 1.26.0)^72^, after which the mean expression level for each gene was determined at each of the four time points. Each row was then centered to mean 0 and scaled to standard deviation of 1. The data was clustered using k-means clustering (k = 6) on a Euclidean distance matrix using the R package TCseq (version 1.10.0)^73^. Cluster number was determined by assessing the stability of the clustering in terms of within-cluster sums of squares over 100 iterations of the clustering. We then determined that the optimal tradeoff between stability and informativeness of each cluster occurred with a cluster number of k = 6. To verify the stability, we ran another 100 random k-means clustering runs using different parameters (nstart = 100, k = 6 and iter.max = 1000). This yielded 100 fully stable clusters that matched very well with the clustering definition maintained in the manuscript (98.3% of genes matched their cluster).

### Comparisons with DICE and biopsy-derived gsTcells data

DE genes from primary naïve CD4+ T cells were retrieved from DICE (https://dice-database.org/)41. Briefly, naïve CD4+ T cells from healthy donors (n = 91) were obtained from blood by FACS and stimulated using aCD3/aCD28 for 4 h. Biopsy-derived gsTcells were obtained from Christophersen et al.^13^ and contained tetramer+ CD4+ T cells and tetramer− CD4+ T cells from CeD patients (n = 5) and gut CD4+ T cells from healthy controls (n = 4). First, we obtained all DE genes that were significant (adjusted *p* value < 0.05) and showed an absolute log2FC > 1. Next, we intersected all DE genes from each dataset to obtain those that were unique per dataset and those that were shared with gsTcells. We then obtained the overlapped DE genes of gsTcells between DICE (n = 1926) and CeD biopsies (n = 144) to evaluate the concordance of those genes using the log2FC. The overlapped genes were divided in four quadrants (Q1–Q4). Q1 and Q3 included concordant DE genes that were upregulated and downregulated, respectively. Q2 and Q4 consisted of non-concordant DE genes, with Q2 being upregulated in gsTcells but not in reference dataset and Q4 being vice-versa.

### Gene set enrichment analysis

Reactome pathways^74^ were used to identify the pathways or biological processes that were enriched for each set of genes. This analysis was performed using the R package clusterProfiler (version 3.14.3)^75^. *p* values were adjusted using the Benjamini–Hochberg procedure to account for multiple testing.

### Quantification and peak calling of DHS sequencing

DHS reads were aligned to hg19 reference genome using bwa (version 0.6.1-r104)^76^ with default settings, after which duplicates were marked using bamUtil (version: 1.0.2)^77^. Alignments were then filtered to have a mapping quality of at least 30 and a primary alignment and to not be duplicated using Samtools (version 1.9)^78^. Peaks were then called using macs2 (version 2.2.6)^79^ enabling –broad –nomodel –shift -125 –extsize 250. Peaks were considered at an FDR threshold < 0.05.

### Differential peak calling of DHS sequencing

To identify differentially accessible sites between timepoints, consensus peaks were first defined using the R package DiffBind (version 2.14.0)^80^, after which raw read counts were determined for each consensus peak. Differentially accessible peaks were then quantified between t0 and t10, t10 and t30, and t30 and t180 using DEseq2 (version 1.26.0)^72^. PC analyses of DHS data were performed on the RPKM-normalized log10-transformed read counts for the consensus peaks. Overlap of peaks between the gsTcells or public datasets was determined with Bedtools multiinter –cluster. TF binding site enrichment was performed with Homer findMotifsGenome.pl with the merged regions of untreated gsTcells as background^81^.

### Enrichment of differentially expressed genes in celiac disease loci

To test for enrichment of DE genes in CeD loci, we used the R package GREA (https://github.com/raguirreg/GREA, version 0.1.0)^19^. We defined CeD genes as genes within a 125 kb window of a CeD GWAS top SNP. We then generated 10,000 random gene-sets that matched the CeD gene-sets in size. The 10,000 random gene-sets were used to generate an empirical null distribution of the overlap between our DE gene-set per cluster and the random gene-sets. We then estimated the one-sided empirical *p *value of the enrichment for each cluster of DE genes.

### Ethical approval

Biological material was obtained from celiac disease patients according to protocols approved by the regional ethics committees (Medical Ethical Committees of VU University Medical Center, Leiden University Medical Center and University of Oslo), and the individuals donating material gave their written informed consent.

## Supplementary Information

Supplementary Information.

Supplementary Figure 1.

Supplementary Figure 2.

Supplementary Figure 3.

Supplementary Figure 4.

Supplementary Figure 5.

Supplementary Figure 6.

Supplementary Figure 7.

Supplementary Figure 8.

Supplementary Figure 9.

Supplementary Figure 10.

Supplementary Figure 11.

Supplementary Figure 12.

Supplementary Table 1.

Supplementary Table 2.

Supplementary Table 3.

Supplementary Table 4.

Supplementary Table 5.

Supplementary Table 6.

Supplementary Table 7.

Supplementary Table 8.

Supplementary Table 9.

## Data Availability

All data (RNA-Seq count tables, DHS counts and peaks) required to reproduce this study have been provided as supplementary files. The raw RNA-Seq and DNAse reads supporting this study are available upon request to the authors as this is privacy sensitive. All code and scripts used to generate the results and figures are available on Github (https://github.com/OlivierBakker/gluten_specific_tcells).
