# Supplementary figures and images for: Potential impact of celiac disease genetic risk factors on T cell receptor signaling in gluten-specific CD4+ T cells
Source: Sci Rep. 2021 Apr 29;11:9252. doi: 10.1038/s41598-021-86612-5 (PMC8085175; doi:10.1038/s41598-021-86612-5)

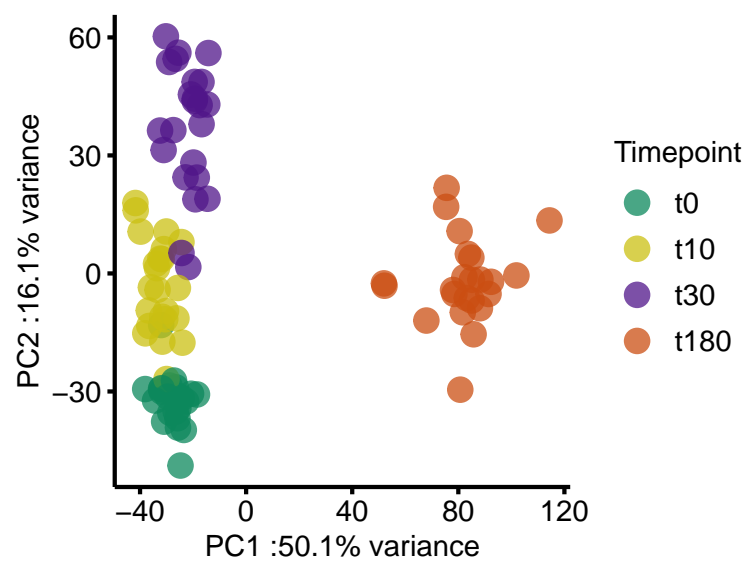

Supplement: Supplementary file 2 — Supplementary Figure 1. [file 41598_2021_86612_MOESM2_ESM.pdf]

A

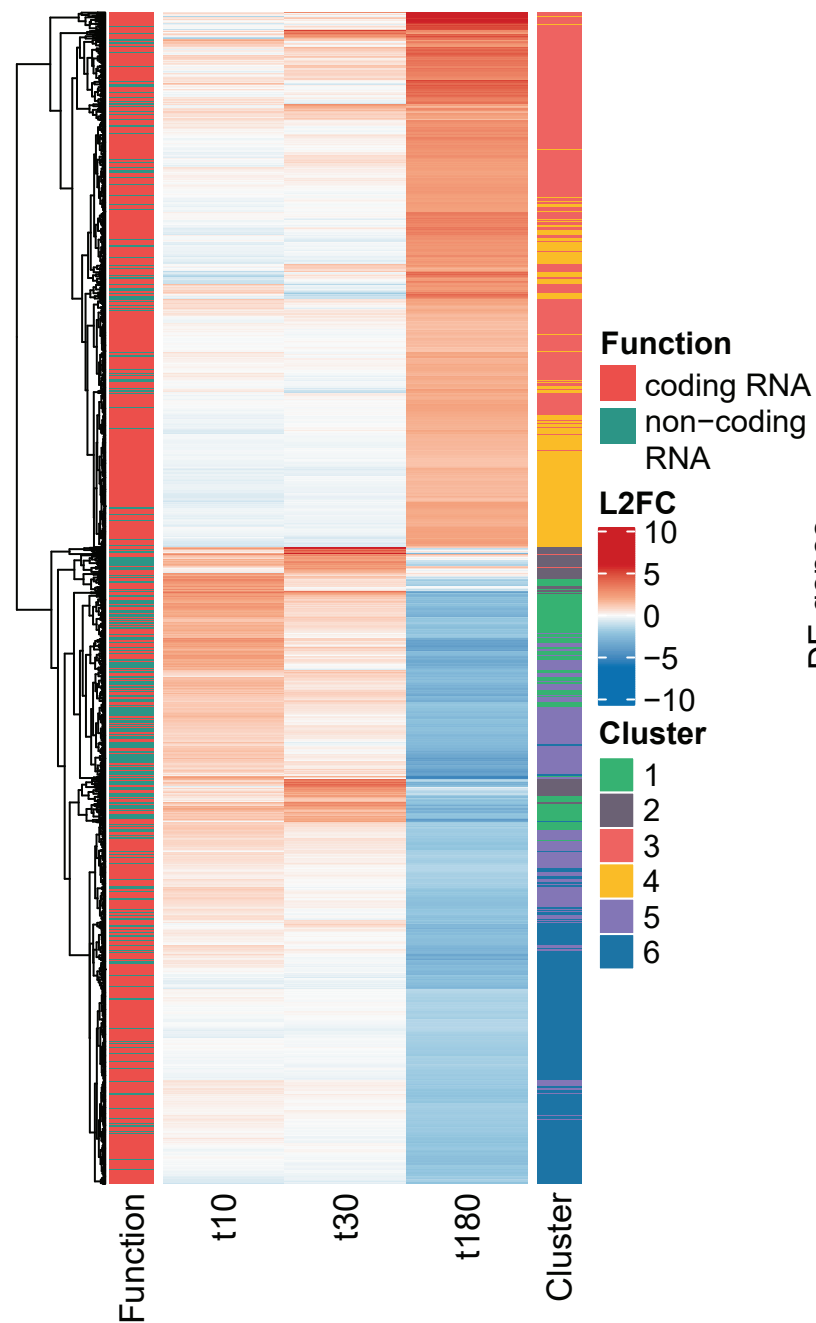

B

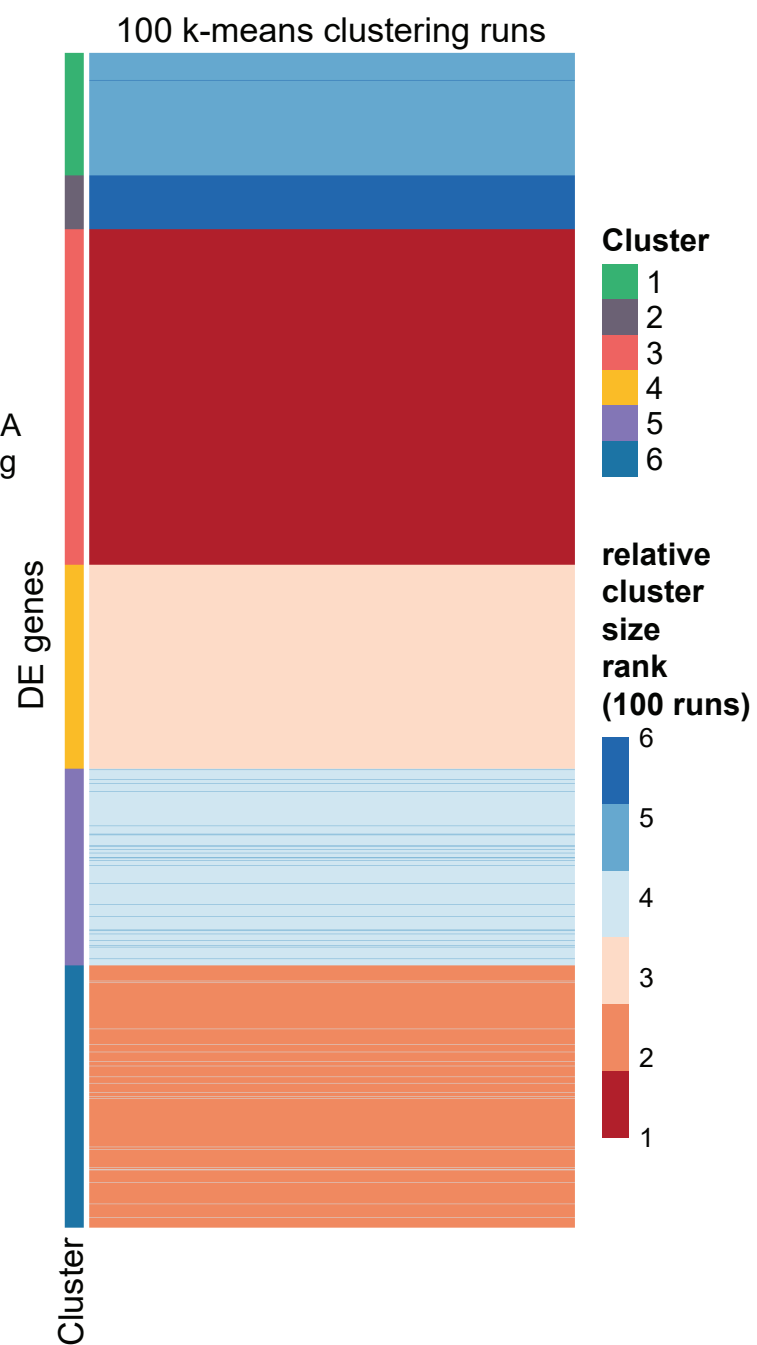

C

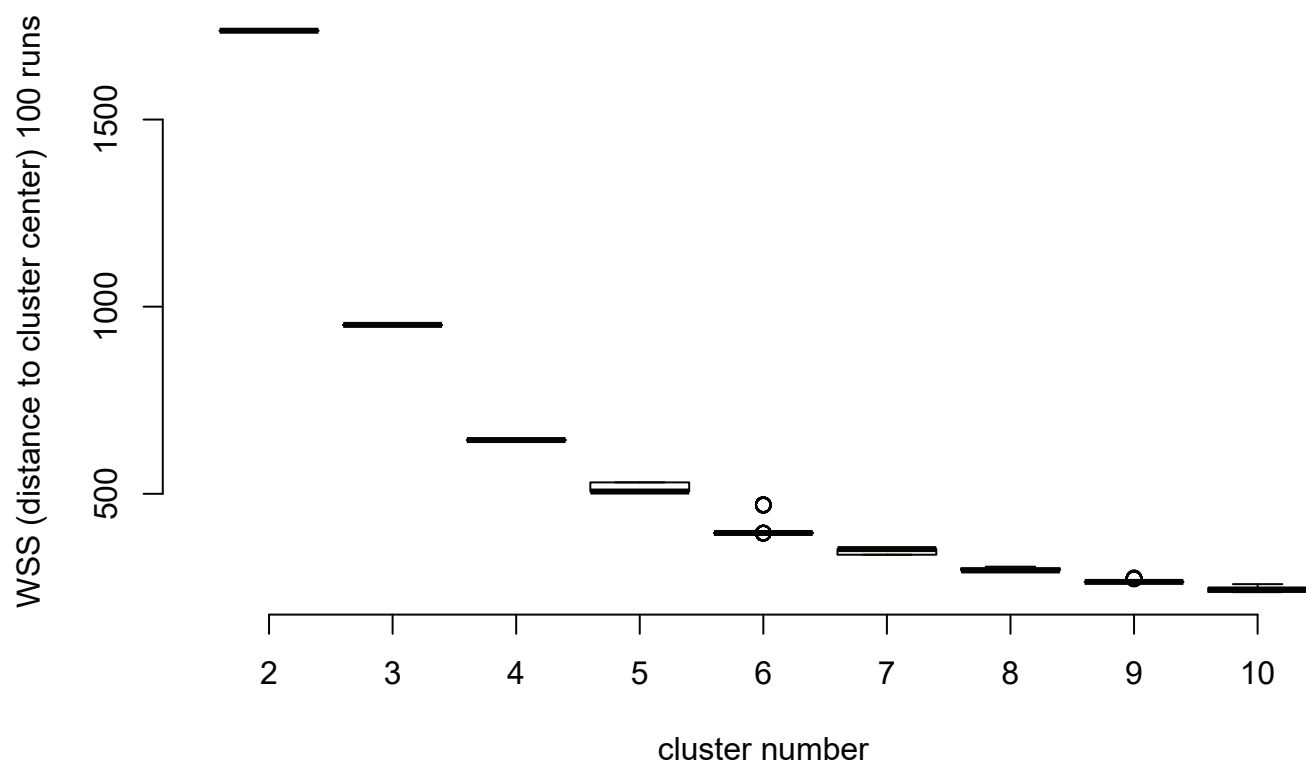

Supplement: Supplementary file 3 — Supplementary Figure 2. [file 41598_2021_86612_MOESM3_ESM.pdf]

A

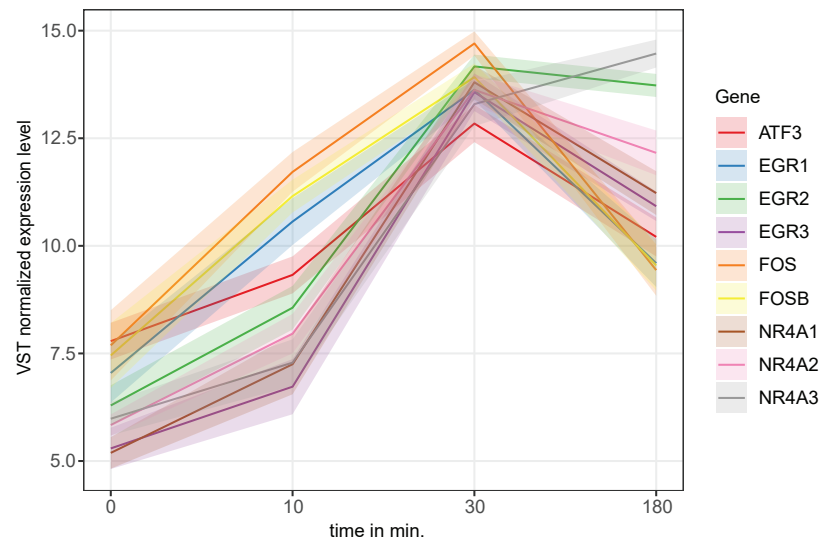

B

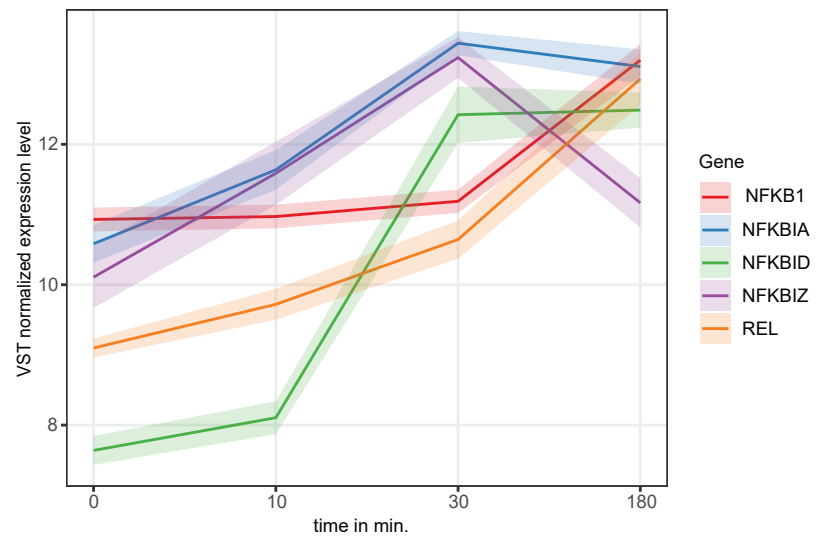

Supplement: Supplementary file 4 — Supplementary Figure 3. [file 41598_2021_86612_MOESM4_ESM.pdf]

A

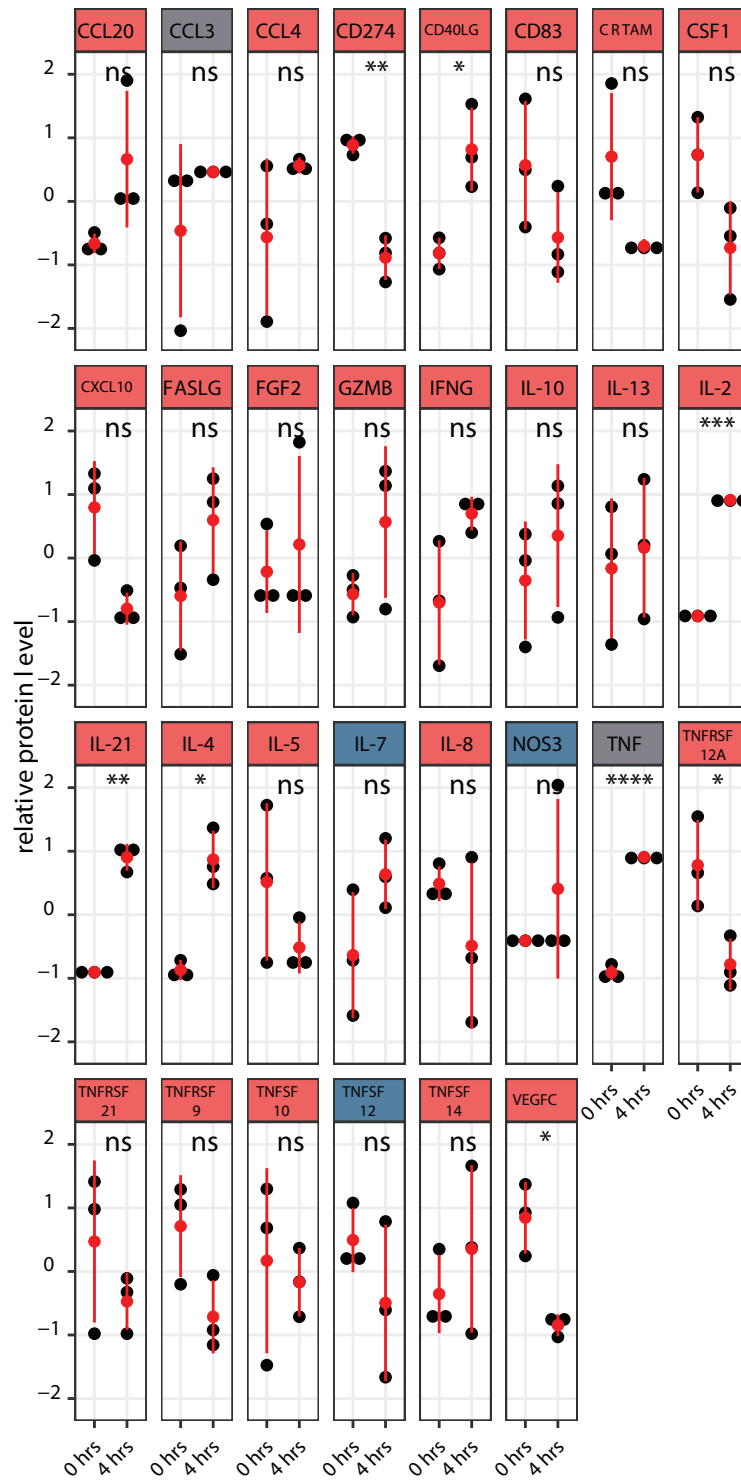

B

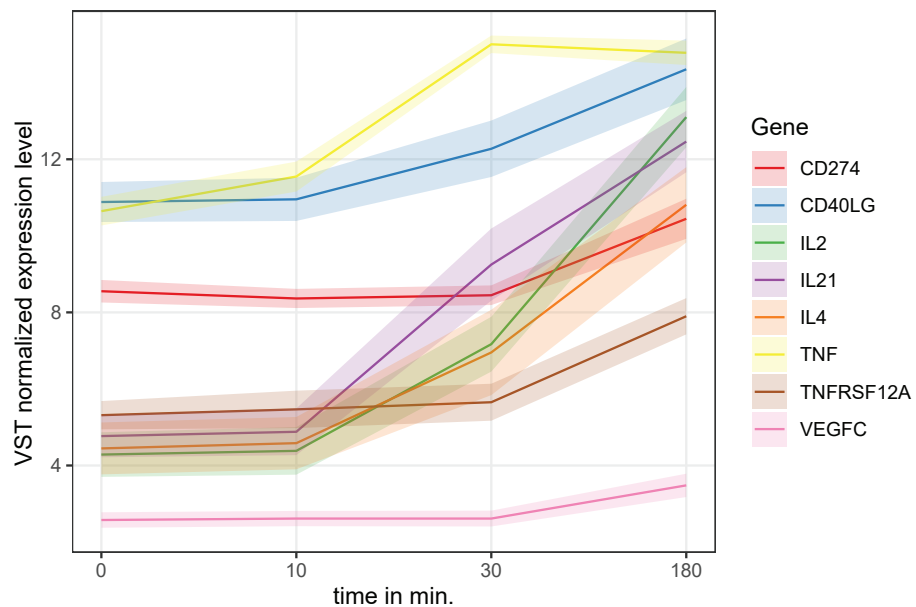

Supplement: Supplementary file 5 — Supplementary Figure 4. [file 41598_2021_86612_MOESM5_ESM.pdf]

A

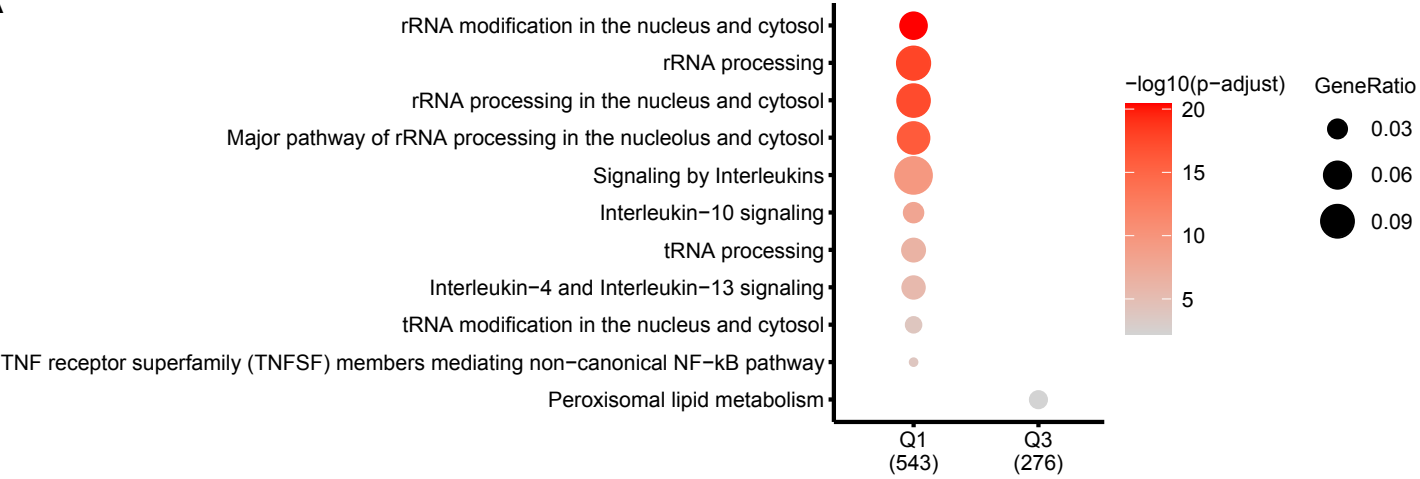

B

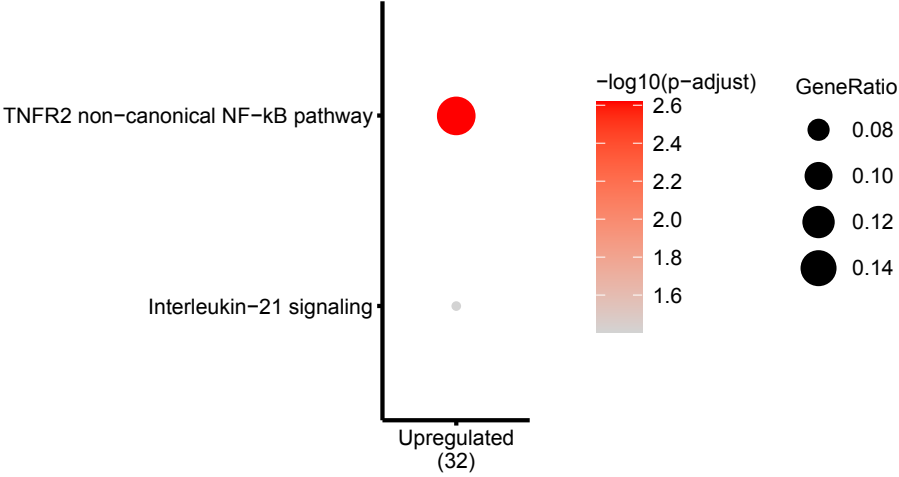

Supplement: Supplementary file 6 — Supplementary Figure 5. [file 41598_2021_86612_MOESM6_ESM.pdf]

A

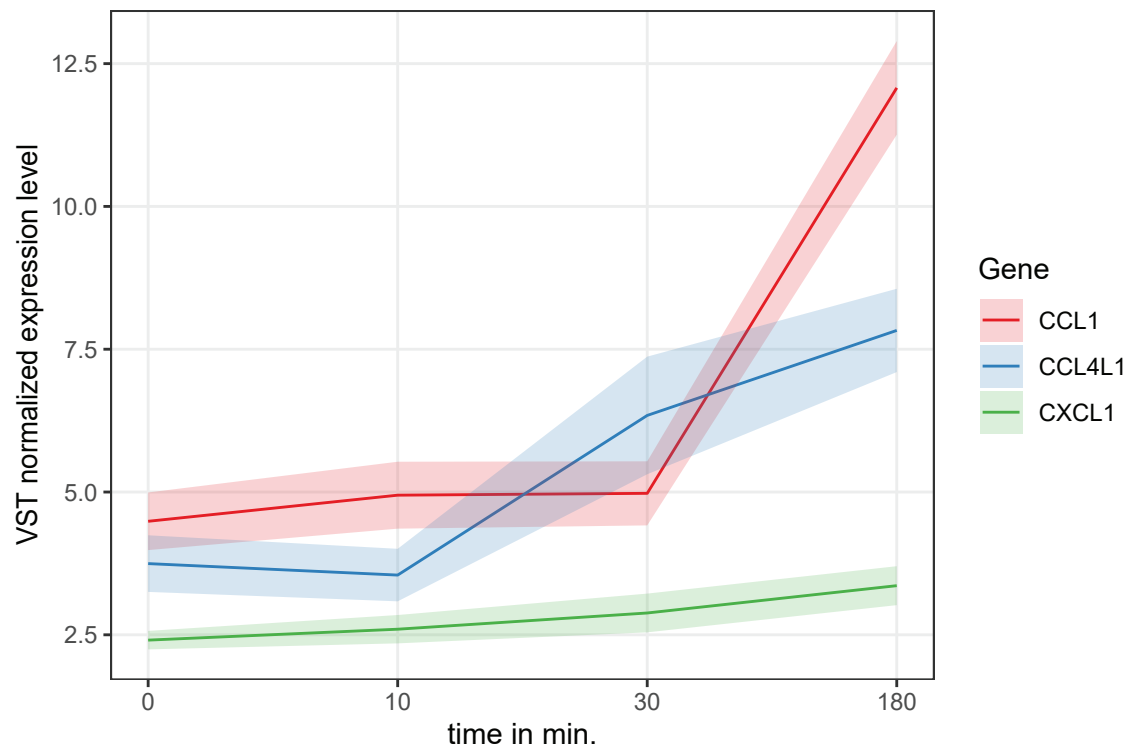

B

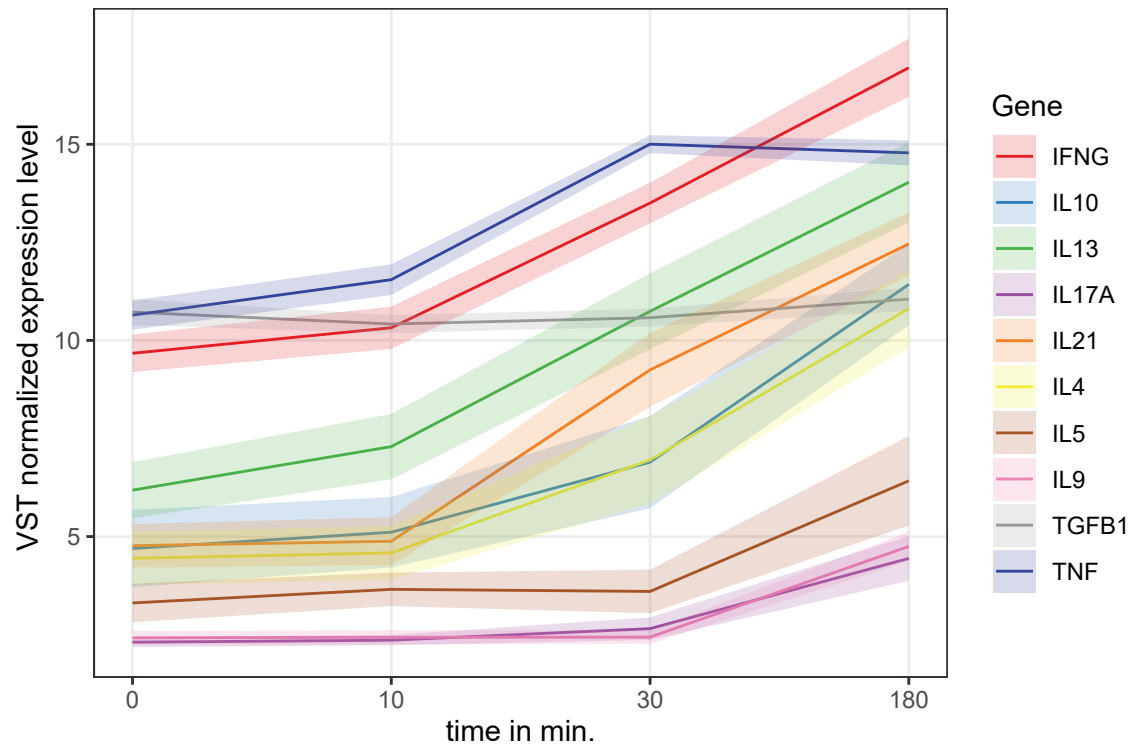

Supplement: Supplementary file 7 — Supplementary Figure 6. [file 41598_2021_86612_MOESM7_ESM.pdf]

A

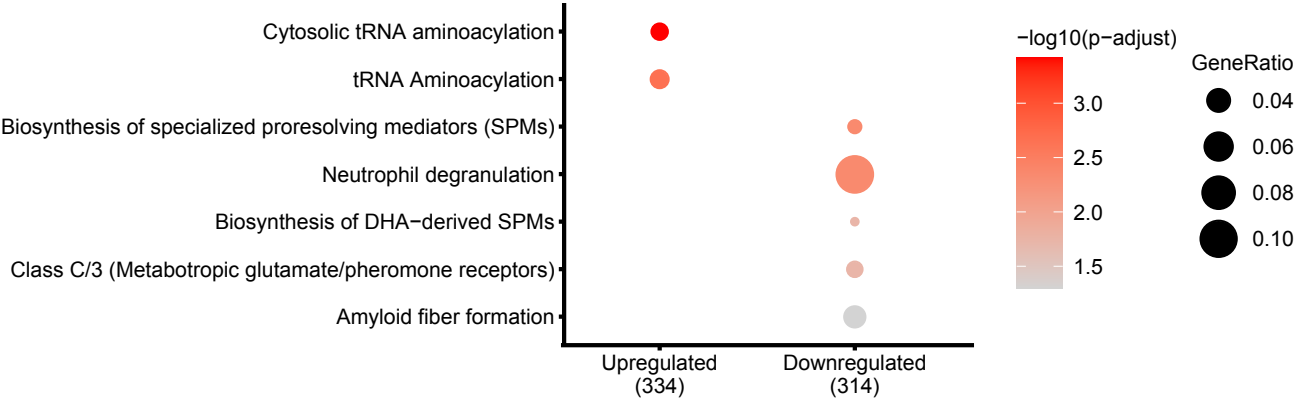

Supplement: Supplementary file 8 — Supplementary Figure 7. [file 41598_2021_86612_MOESM8_ESM.pdf]

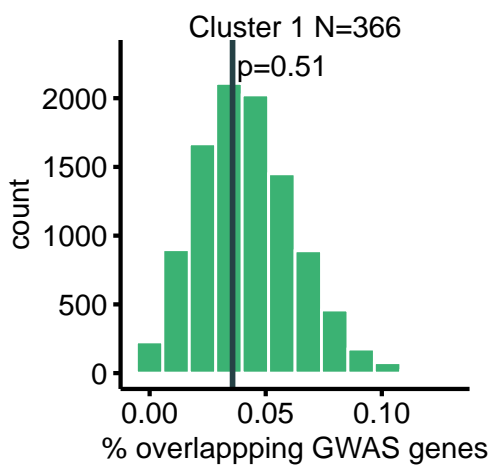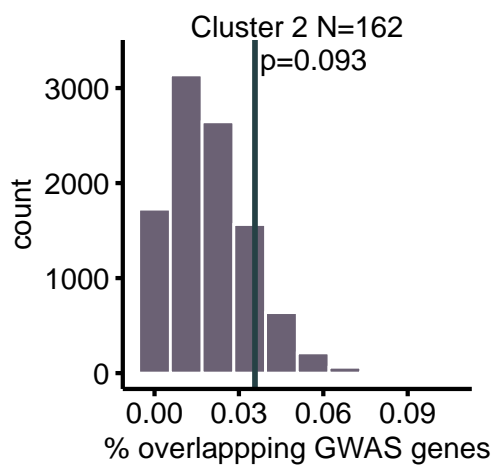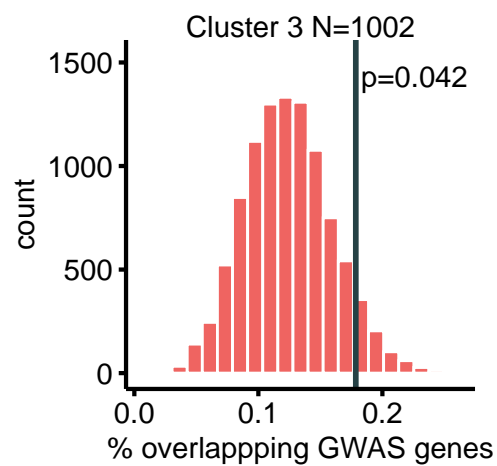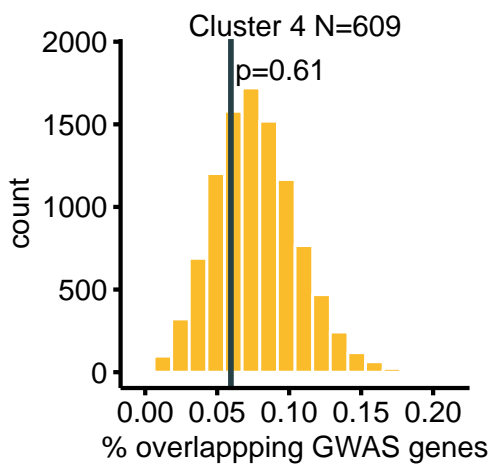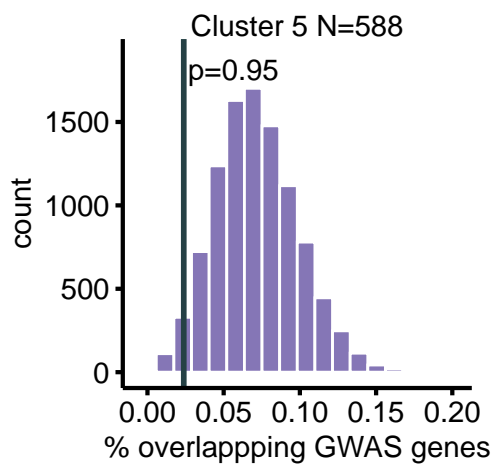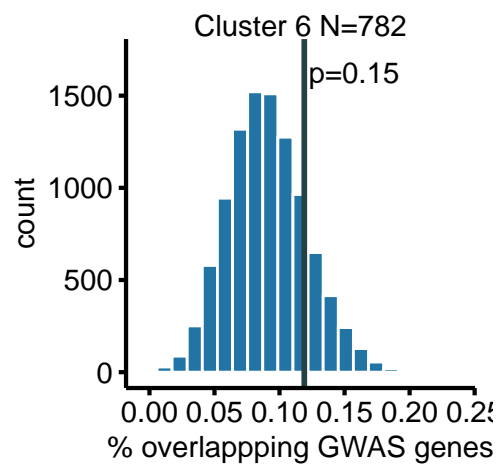

Supplement: Supplementary file 9 — Supplementary Figure 8. [file 41598_2021_86612_MOESM9_ESM.pdf]

A

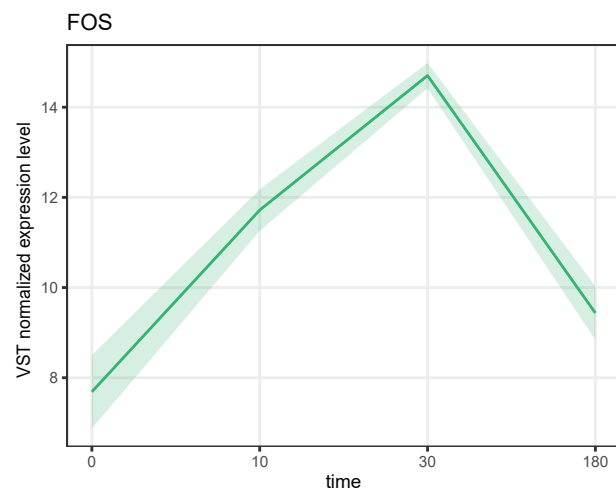

B

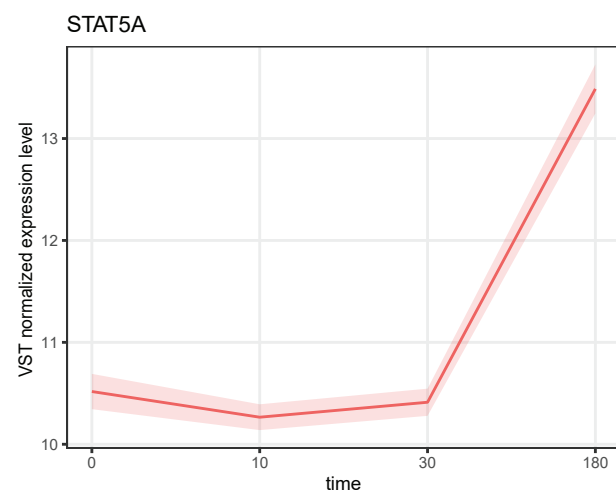

C

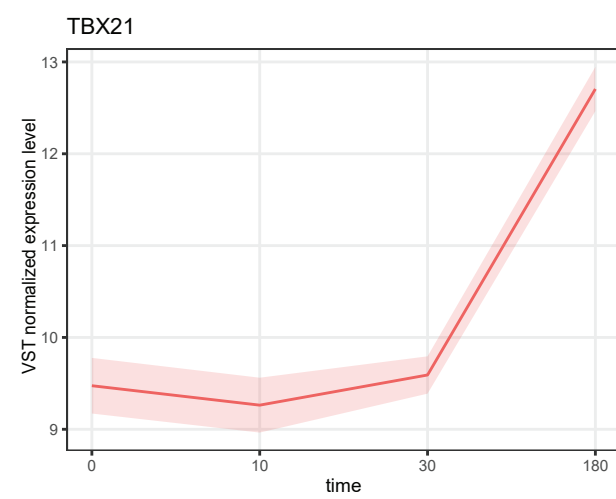

Supplement: Supplementary file 10 — Supplementary Figure 9. [file 41598_2021_86612_MOESM10_ESM.pdf]

A

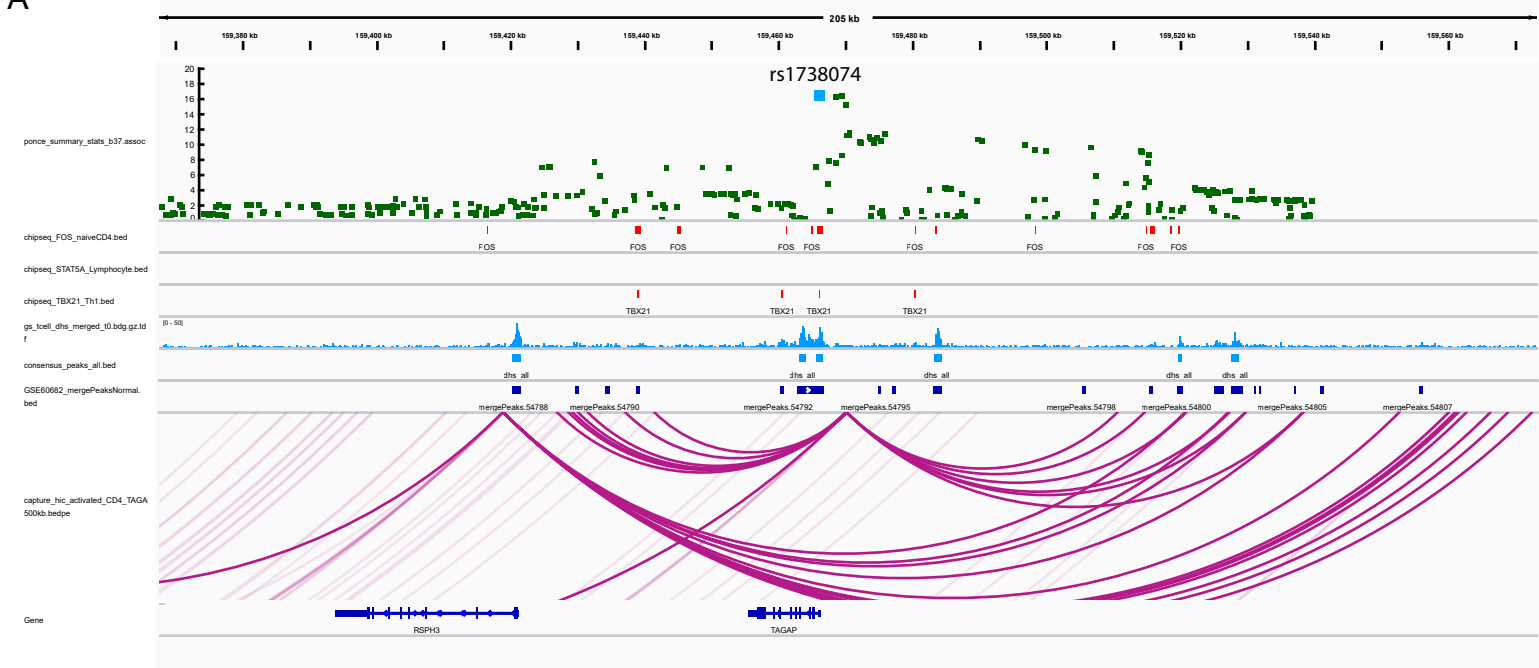

B

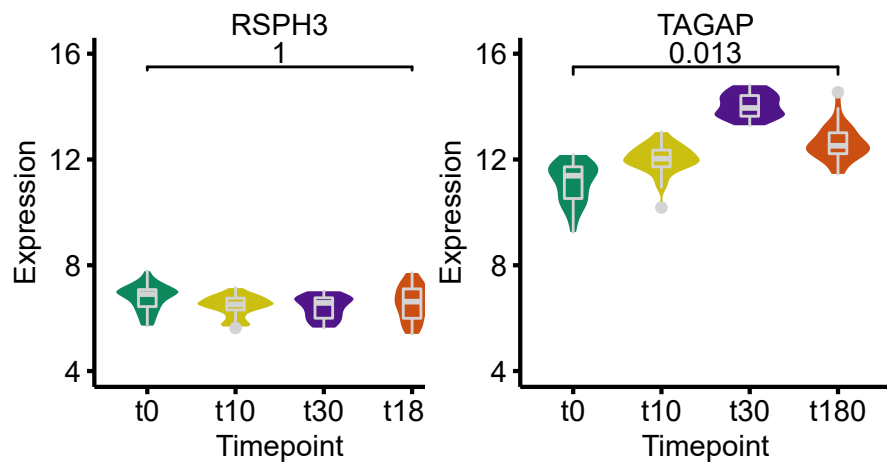

Supplement: Supplementary file 11 — Supplementary Figure 10. [file 41598_2021_86612_MOESM11_ESM.pdf]

A

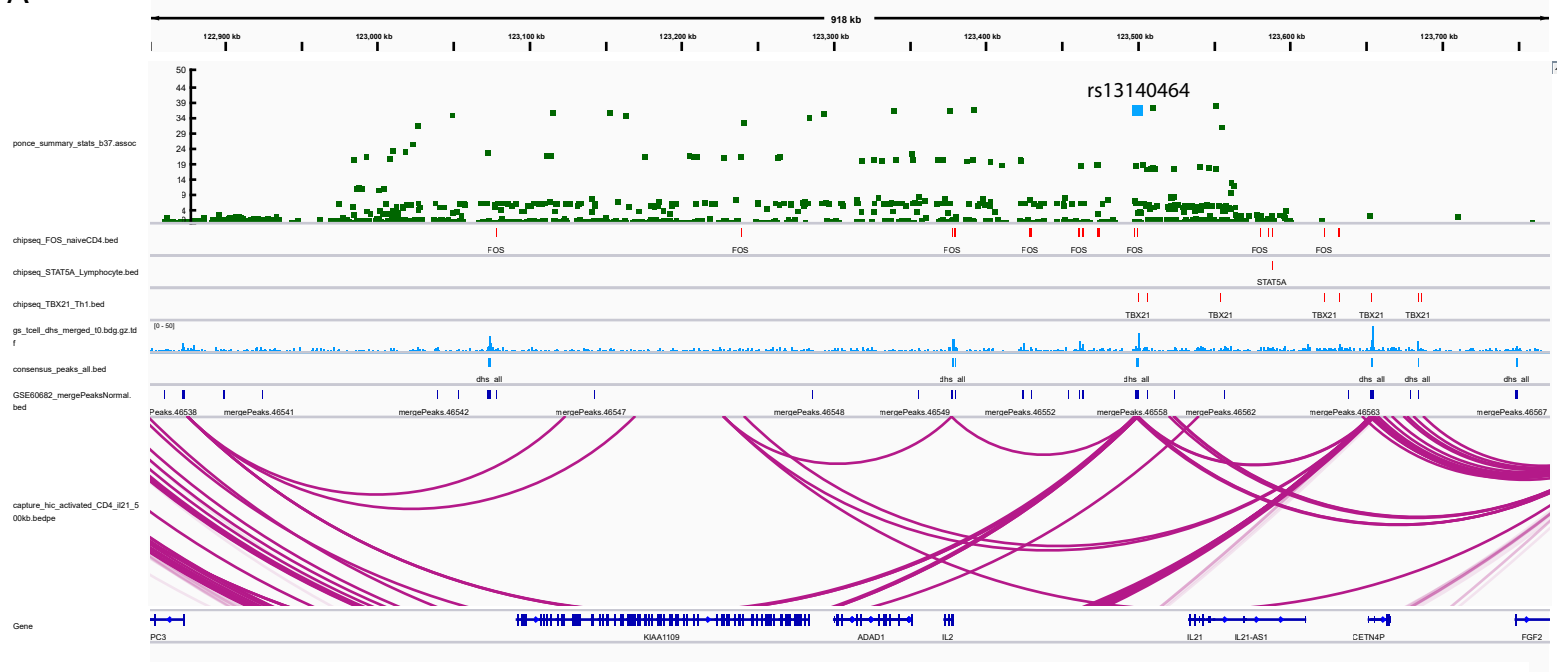

B

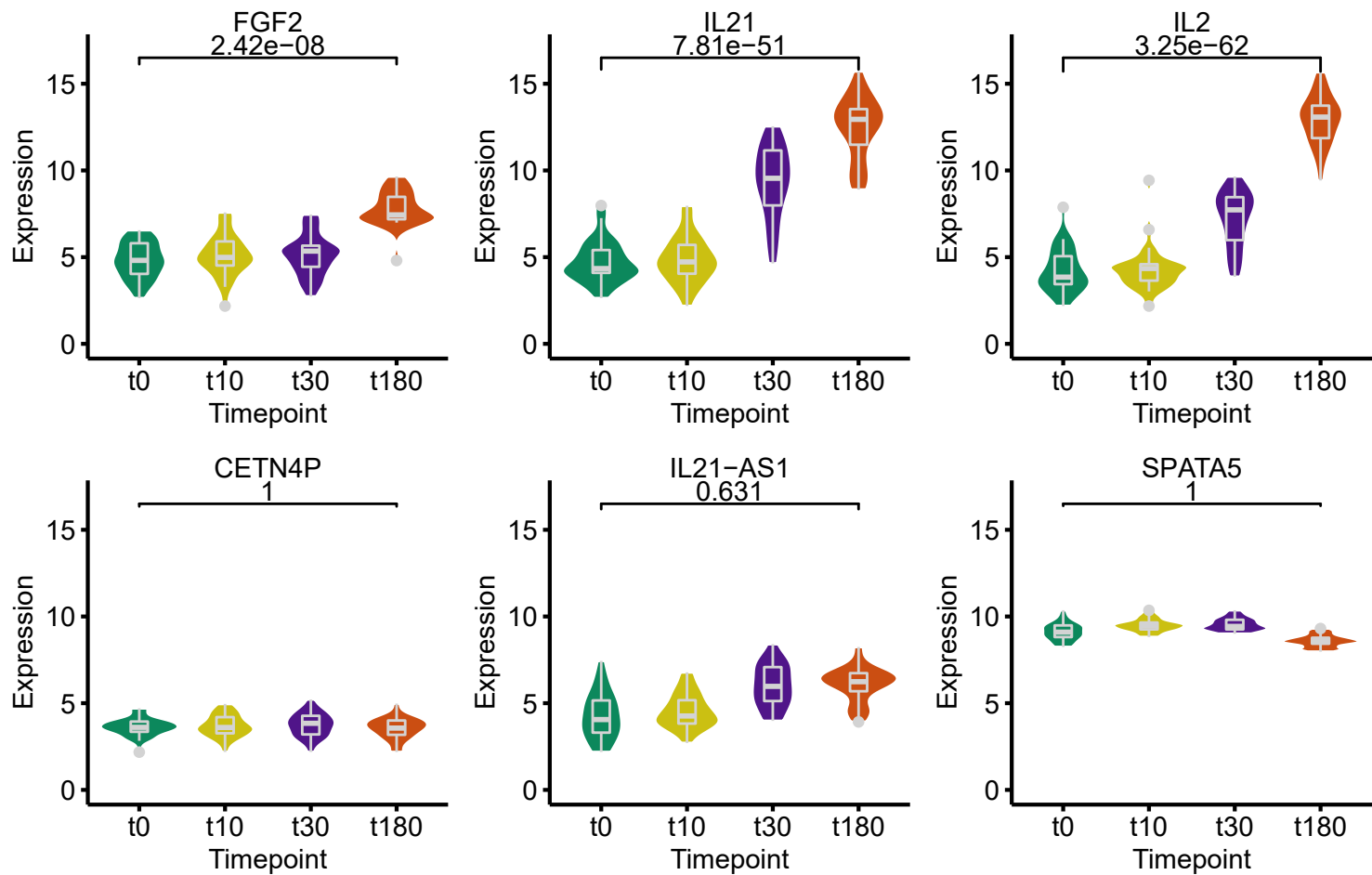

Supplement: Supplementary file 12 — Supplementary Figure 11. [file 41598_2021_86612_MOESM12_ESM.pdf]

A

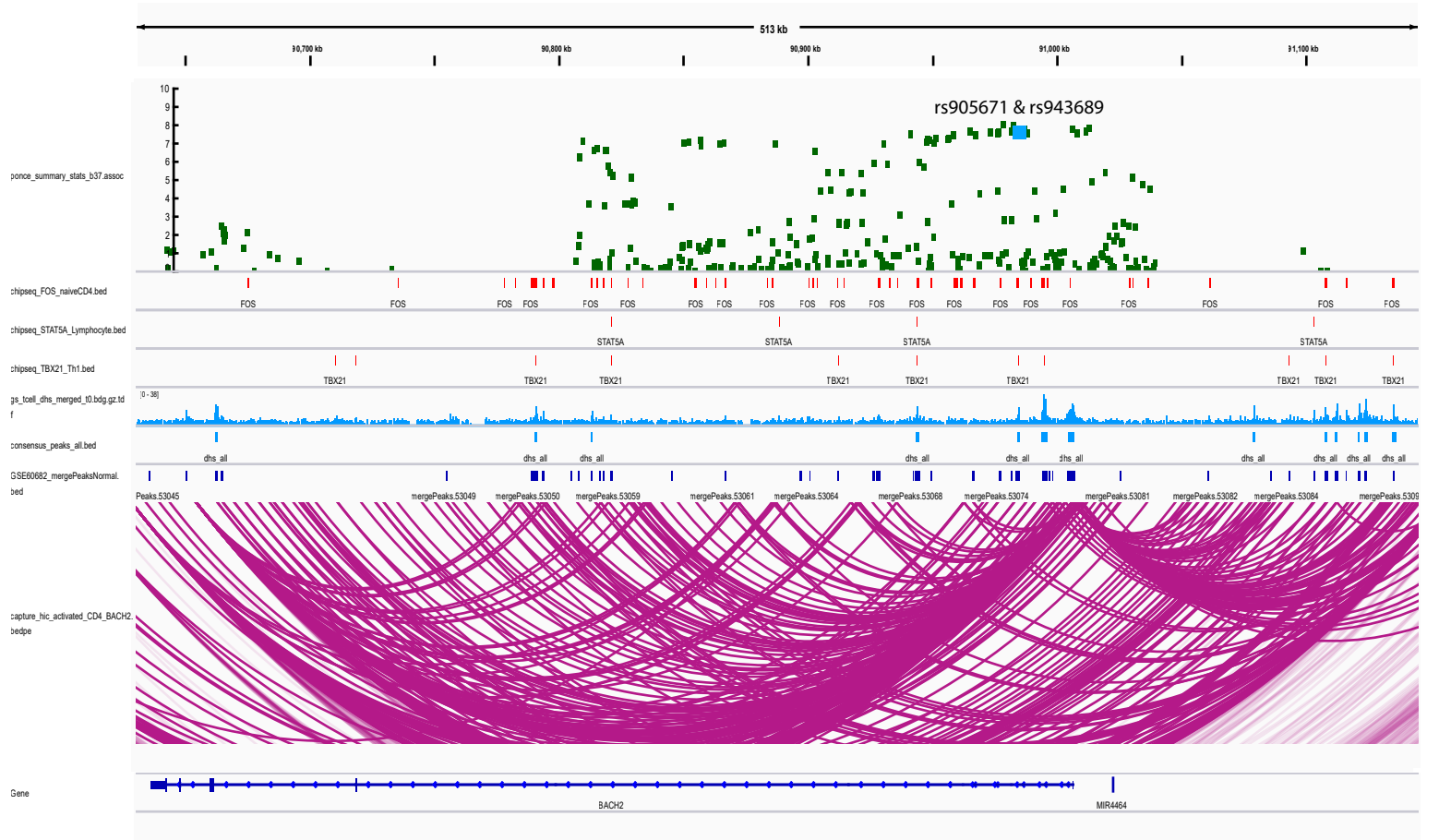

B

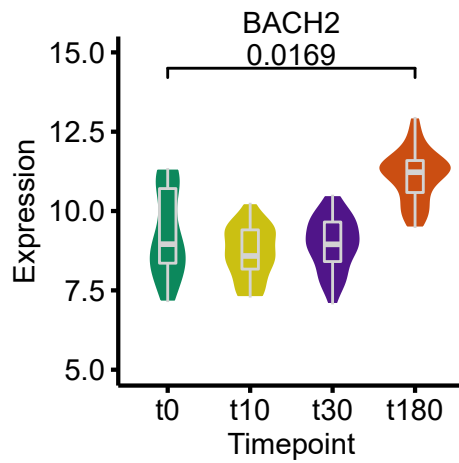

Supplement: Supplementary file 13 — Supplementary Figure 12. [file 41598_2021_86612_MOESM13_ESM.pdf]
